# An Integrated Spectroscopy–Microkinetic Approach to Identifying Active‐Site Distributions in Palladium Single‐Atom Catalysts

**DOI:** 10.1002/cssc.71026

**Published:** 2026-09-07

**Authors:** Ronan Gleeson, Alessandro Fortunelli, Mathias Meidahl Hommelgaard, Mauro Stener, Yang Hu, Heine Anton Hansen

**Affiliations:** ^1^ Department of Energy Conversion and Storage Technical University of Denmark Kongens Lyngby Denmark; ^2^ CNR‐ICCOM Consiglio Nazionale delle Ricerche Pisa Italy; ^3^ Dipartimento di Scienze Chimiche e Farmaceutiche University of Trieste Trieste Italy

**Keywords:** density functional calculations, electrochemistry, kinetics, photoelectron spectroscopy

## Abstract

Single‐atom catalysts based on metal‐doped carbonaceous materials are promising catalysts. However, the synthesis procedure dictates the local atomic environment, often resulting in a heterogeneous distribution of active sites with distinct properties. Here, we identify these active sites and quantify the ensemble effect of single sites on the activity and selectivity for the oxygen reduction reaction (ORR) of a palladium/nitrogen‐doped‐graphene single‐atom (Pd–N–C) catalyst. By combining core‐level spectroscopic data from literature with in‐house DFT calculations and microkinetic modeling, we screened various local active site structures. A thermodynamic formation analysis complemented by XANES exclude edge and porous sites based on their poor agreement with experiment and energetics respectively. The remaining basal site distribution within the model’s assumptions is best‐fit quantified using absolute XPS calculations revealing a site distribution dominated by basal plane PdN_4_C_10_ (67%–51%) and nitrogen enriched PdN_4_C_10_ + 1N (49%–33%) active sites. The origin of stability for basal structures is geometrically rationalized by the trend in the number of stabilizing hexagonal rings. According to microkinetic ORR rate analysis, the onset potential for both basal active sites is governed by the initial hydrogenation step, whilst nitrogen enrichment increases activity but reduces H_2_O_2_ selectivity.

## Graphical abstract

By integrating core‐level imaging with electrochemical testing, we reveal active site distributions that remain obscured when using these methods in isolation. We demonstrate that understanding the transition from independent site activity to ensemble performance is crucial for better agreement with experiment and overall catalyst design.

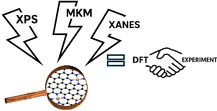

## 1 Introduction

The research and development of effective energy storage and conversion solutions are essential for reducing society’s dependence on fossil fuels. The electrochemical transformation of oxygen has been of particular importance, with the production of value‐added, decentralized hydrogen peroxide [1], aiming to reduce societal reliance on the resource‐intensive anthraquinone‐based approach. The oxygen reduction reaction (ORR) requires novel energy solutions, such as proton exchange membrane fuel cells (PEMFCs) and rechargeable metal‐air batteries [2, 3], to optimize the thermodynamics and kinetics of product formation. The ORR kinetics of the cathode are particularly sluggish, with inefficiencies reaching values nearly an order of magnitude higher than those of the anode material. Industrial PEMFCs are highly dependent on the use of precious metal nanoparticles to overcome the poor cathode kinetics, albeit resulting in high development costs (≈50% of the total PEMFC cost) and high precious metal loadings. Nanoparticle systems also favor the four‐electron pathway, selectively producing H_2_O over H_2_O_2_ [4]. In contrast, the H_2_O_2_ forming two‐electron pathway can be promoted in acidic media through the use of atomically dispersed metal/nitrogen carbon‐based (M–N–C) catalysts—more commonly known as single‐atom catalysts (SACs) [5]. M–N–C solutions can be synthesized at a fraction of the metal loading cost of nanoparticles, ensuring a high utilization frequency and promotion of highly selective and active H_2_O_2_‐producing sites. However, significant challenges are still posed toward characterizing the true active sites in M–N–Cs.

A number of studies have hypothesized various likely active sites, the most probable being the metal‐porphyrin‐like pyridinic configuration MN_4_C_10_ [6, 7, 8, 9, 10, 11, 12, 13], with disordered microporous pyrrolic MN_4_C_12_ [12, 13] and MN_4_C_8_ [8, 14] sites also proposed in literature. Additionally, zigzag (Z) and armchair (A) edge active sites have been proposed as thermodynamically stable under synthesis conditions [15, 16, 17, 18, 19]. Due to the broad landscape of potential active sites, relating experimental structure to a single active site or a distribution of active sites is nontrivial. Furthermore, the agglomeration of metal atoms with the consequent formation of nanoclusters further obfuscates accurate active site characterization [20]. Understanding the composition and distribution of active sites is therefore critical as these parameters govern the emergent catalytic activity and selectivity of the material.

Many key studies have achieved major improvements in the performance of M–N–C catalysts, yet most share a common assumption: the active site configuration is assumed in the study. Instead, one should characterize the material directly through a combination of experimental and in silico analyses, preferably based on first‐principles methods. Although experimental characterization methods are well established, the integration of first‐principles simulation with experiments is still absent in many M–N–C characterization studies. Zitolo et al. [13] proposed a framework that computes such a distribution of M–N–Cs using a first‐principles approach integrated with experimental data. The distribution was calculated using a spin density analysis and an X‐ray absorption near edge structure spectroscopy (XANES) screening procedure. A machine learning approach has similarly been used to predict the distribution of active‐site configurations in cobalt SACs for the hydrogen evolution reaction [21].

The present study applies a modified version of the framework established by Zitolo et al., retaining the XANES‐based screening while complementing it with a quantitative X‐ray photoelectron spectroscopy (XPS) approach. This study’s unique alternative approach quantifies the distribution of active sites by comparing experimental and computed absolute XPS spectra. XPS core‐level shifts can be determined with high precision both experimentally and from first‐principles simulations, enabling quantitative comparison between the two. To quantify the accuracy of the density functional theory (DFT) self‐consistent‐field (ΔSCF) method for the calculation of absolute XPS binding energies [22, 23], a validation study of small molecules followed by nitrogen‐doped graphene is first presented. The new framework then begins with an initial screening of candidate structures by thermodynamic formation energy, complemented by comparison of experimental and computed XANES spectra. The validated ΔSCF approach is used thereafter to quantify a distribution of active sites according to their absolute XPS binding energy and optimized intensities relative to experiment. Sensitivity analysis is also performed to determine a physically realistic range of the calculated distribution due to errors in the DFT binding energy calculations and/or peak fitting. A Boltzmann population analysis of Gibbs thermodynamic formation energies is performed to support the spectroscopically quantified distribution. The proposed framework is applied to an experimental palladium‍/‍nitrogen‐doped‐graphene single‐atom (Pd–N–C) material found in literature [24], for which the active site distribution and performance are established thereafter. This preliminary work concentrates solely on Pd active sites, which will aid our future studies on other ORR relevant metals such as Co, Ni, and Fe.

Typically, the in silico performance of a catalyst is evaluated using a single active site, which is often too simplistic due to the heterogeneity of atomically dispersed materials. The present framework will provide insight into what the “other” active site found alongside the ubiquitous pyridinic site—a question that is commonly posed by M–N–C studies [12, 19]. Furthermore, a microkinetic model provides physical insight into the origin of the ORR activity and selectivity of single sites and their ensemble site performance. The degree of single‐site mixing used in the model is determined by the distribution of sites calculated from core‐level spectroscopy. A microkinetic rate analysis will give insight into which are the potential‐dependent rate‐controlling steps along the working potential range. The microkinetic model also checks whether the calculated postsynthesis core‐level spectroscopy distribution of active sites remains compatible with the electrochemical response within the assumed energetic perturbations. This framework establishes a first‐principles description of the active site structure, yielding a more accurate representation of site heterogeneity and offering physical insight into how individual sites contribute to the catalyst’s overall performance.

## 2 Methods

### 2.1 Active Site Distribution Framework

**SCHEME 1:**
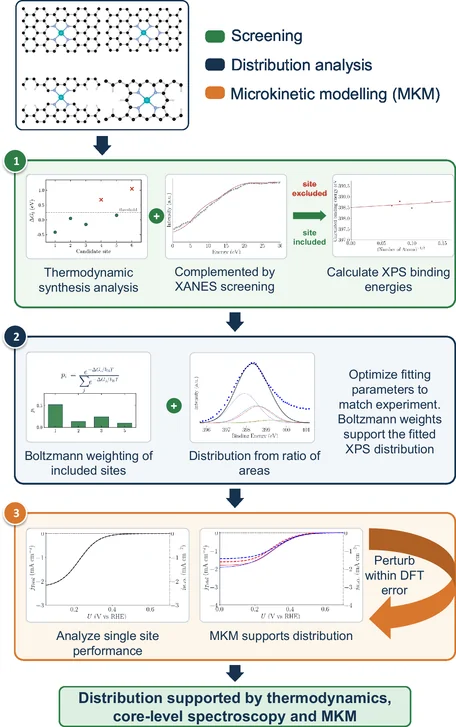
(1) The workflow begins by screening a pool of active sites based on satisfying the thermodynamic criterion, Δ*G*_f_ ≤ 0 eV. A small XANES *R*‐factor provides a complementary sanity check on the thermodynamic analysis. Structures that pass this screening undergo an extrapolated XPS procedure to calculate their characteristic absolute binding energies. (2) A fitting of each binding energy is performed, and the distribution of active sites is quantified with reference to an experimentally deconvoluted XPS spectrum. A Boltzmann weighting of the thermodynamic formation energies aims to support the spectroscopically determined distribution. (3) A microkinetic model is used to assess the distribution compatibility between core‐level spectroscopy measurements and operation within plausible uncertainty. Intermediate Gibbs free energies are allowed to be perturbed within the typical error (0.2 eV) of DFT functionals to achieve a better fit. Microkinetic modeling also provides physical insights into the performance of individual active sites and the ensemble.

In the following section, the computational aspect of the framework is presented, highlighting the framework’s ability to identify and quantify the active site composition of an experimental sample. A particular emphasis will be placed on detailing the computational aspect of the framework as it involves numerous nontrivial steps that the reader may not be familiar with. In contrast, the extraction of the equivalent experimental information has been extensively established in previous studies. A schematic of the individual steps of the framework is given in Scheme 1.

#### 2.1.1 Calculate XANES and Thermodynamic Screening Cut‐Off

The initial XANES step begins by calculating the XANES spectrum of each initial structure from first principles using the Finite Difference Method Near Edge Structure (FDMNES) program [25, 26, 27]. Other programs can be used, but only the FDMNES program has been validated for use in this framework. The FDMNES program uses the muffin‐tin approximation, or typically, the higher‐level finite difference scheme, to calculate the XANES spectrum. On modern high‐performance computing architecture, calculating finite difference spectra is relatively inexpensive. Perturbations in coordination numbers, bond distances, and/or disorder parameters are permitted within a certain threshold to improve fit with experiment. The *R*‐factor is used to determine the goodness of fit with XANES experiment, as given by

(1) $$R=\frac{\sum_{i}{\left[\mu_{\exp}\;(E_{i})-\mu_{\mathrm{calc}}\;(E_{i})\right]}^{2}}{\sum_{i}{\left[\mu_{\exp}\;(E_{i})\right]}^{2}}$$

where *μ*_exp_ is the experimental absorption intensity and *μ*_calc_ is the calculated absorption intensity at each energy *E*_*i*_. Structures proceed from the initial screening step based on a favorable thermodynamic (Δ*G*_f_  ≤ 0 eV) synthesis energy. A small XANES *R*‐factor provides a complementary sanity check on the thermodynamic analysis. Within this framework, the following thermodynamic formation energy Δ*G*_f_ with respect to graphene is used

(2) $$\begin{array}{cc} \Delta G_{f} & =\;E(M_{a}N_{b}C_{c}H_{d})-E(C_{e})\\ & \;+\;(e-c)\mu_{C}-a\mu_{M}-b\mu_{N}-d\mu_{H} \end{array}$$

where *E*(*M*_a_ *N*_b_ *C*_c_ *H*_d_) and *E*(*C*_e_) are the DFT energies of the M–N–C site and graphene sheet, respectively. *μ*_C_ is the chemical potential of a carbon atom, defined as the DFT energy per carbon atom in graphene. *μ*_M_ is the chemical potential of a single metal atom (i.e., Pd) calculated using DFT. *μ*_N_ is the chemical potential of nitrogen defined using the DFT‐calculated free energy of NH_3_, H_2,_ and N_2_ at room temperature and 1 bar of pressure.

(3) $$\mu_{N}\;\left(n\right)=n\left[G\;\left({\mathrm{NH}}_{3}\right)-\frac{3}{2}G\;\left(H_{2}\right)\right]+\frac{1}{2}[\left(1-n\right)\;G\;\left(N_{2}\right)]$$

here *n* > 2 represents a high content of NH_3_—which is typical for syntheses promoting M–N–C structure formation at temperatures greater than 500 °C [28]. As *n* is not a quantifiable parameter, it is treated as an adjustable parameter. *μ*_H_ is the chemical potential defined as the free energy per atom of hydrogen in H_2_. The effect of synthesis temperature can be accounted for using vibrational entropy and zero‐point energy (ZPE) corrections derived from experimental gas‐phase heat capacity data [29].

#### 2.1.2 Calculate XPS Binding Energy

Each acceptable active site is transformed to a cluster model, where the relevant absolute XPS binding energy is calculated using an extrapolated ΔSCF approach. The all‐electron Amsterdam Density Functional (ADF) [30] program is chosen for its robust convergence of core–hole calculations—aided by a Foster‐Boys localization scheme [31] that prevents variational collapse during SCF cycles. This is especially necessary for the convergence of hybrid functional calculations. ADF performs calculations using a Slater‐type basis [32], which helps to correctly describe the nuclear cusp behavior in the core region—critical for accurate core–hole calculations. A frozen‐core scheme is used to reduce the associated computational cost for heavy elements in a controlled manner. With regard to defining the absolute XPS binding energy, two reference schemes are presented, both serving to validate the consistency of the calculated XPS energy. The valence band maximum (VBM) method references the highest occupied orbital [22, 23], defining the VBM binding energy as

(4) $$E_{B}^{\mathrm{VBM}}=E_{N-1}^{\mathrm{ch}}-E_{N-1}^{\mathrm{gs}}$$

where $E_{N-1}^{\mathrm{ch}}$ is the core–hole energy and $E_{N-1}^{\mathrm{gs}}$ is the energy required to remove an electron from the highest occupied state of the ground state system. The *ɛ*_max_ reference scheme [33, 34] references the ground state energy $E_{N}^{\mathrm{gs}}$ and the Kohn–Sham eigenvalue of the highest occupied state *ɛ*_max_, giving a binding energy as

(5) $$E_{B}^{{ɛ}^{\max}}=E_{N-1}^{\mathrm{ch}}-E_{N}^{\mathrm{gs}}-{ɛ}_{\max}$$

It has been shown that in the limit of a dilute hole gas, Equations (4) and (5) converge, although convergence rates can differ between the two approaches [34]. In order to transform the XPS binding energy from a cluster basis to an infinite basis, one must linearly extrapolate with respect to $\frac{1}{\sqrt{N}}\;\to \;0$

(6) $$\begin{array}{cc} E_{\text{cluster}}\;(N) & =E_{\text{XPS}}+\frac{m}{\sqrt{N}} \end{array}$$

(7) $$\begin{array}{cc} E_{\text{XPS}} & =\lim_{N\to \infty }E_{\text{cluster}}\;(N) \end{array}$$

where *N* is the number of atoms. Therefore, *E*_XPS_ is considered the XPS binding energy that most accurately represents the experimental sample. For reference, the gas‐phase binding energy is defined as

(8) $$E_{B}^{\mathrm{gas}}=E_{N-1}^{\mathrm{ch}}-E_{N}^{\mathrm{gs}}$$

where $E_{N}^{\mathrm{gs}}$ is the ground state energy of the system.

#### 2.1.3 Calculate Site Distribution

For each acceptable active site that satisfies the criterion, Δ*G*_f_ ‍ ≤  0 eV, a Gaussian peak centered at the calculated extrapolated XPS binding energy is optimized until the summation of all peaks fits the experimental peak of interest. A Lorentzian, Voigt (convolution of a Gaussian and a Lorentzian function), etc. fitting could instead be employed if the function better represents the experimental line shape. In practice, the peak amplitude *A*_*i*_ of each Gaussian *i* is optimized such that the sum of all contributions reproduces the experimental spectrum, given as

(9) $$G_{\mathrm{calc}}=\sum_{i=1}^{N}A_{i}\;\exp\left[-\frac{{\left(x-\mu_{i}\right)}^{2}}{2\sigma_{i}^{2}}\right]\approx G_{\exp}$$

where *x* is the absolute binding energy (eV) obtained from the ΔSCF calculations, *μ*_*i*_ is the mean, and *σ*_*i*_ is the peak width. *σ* can be treated as a fitted parameter, or taken directly from the experimental spectrum. A sequential least squares programming (SLSQP) optimizer from SciPy [35] is used to minimize the residual sum of squares between experimental and predicted model values. To avoid convergence to a local minima, random initial guesses are initialized ensuring a broad sampling of the parameter space. The individual Gaussian peak areas are then taken as a ratio of each other, thus representing a distribution of active sites.

As a thermodynamic check, the computed core‐level spectroscopy distribution is compared to a weighted Boltzmann population ensemble (*P*) of the thermodynamic Gibbs formation energies (Δ*G*_f_) for each species *i*

(10) $$P\;\left(i\right)=\frac{\exp\;\left(-\frac{\Delta G_{f}\;(i)}{k_{B}T}\right)}{\sum_{j=1}^{M}\exp\;\left(-\frac{\Delta G_{f}\;(j)}{k_{B}T}\right)}$$

where *k*_B_ is the Boltzmann constant and *T* is the synthesis temperature.

#### 2.1.4 Assess Distribution and Analyze Site Performance

If accessible, a microkinetic model can check whether the distribution of active sites remains compatible between postsynthesis ex situ core‐level spectroscopy and operando electrochemical measurements—within the typical error of DFT functionals. This is achieved by summing the individual calculated active site polarization curves to fit the experimental polarization curve. To improve the fit, DFT energies can be perturbed by up to 0.2 eV, which is the typical error in DFT functionals. As discussed previously in‐depth [36], microkinetic modeling can be extremely sensitive to DFT errors and approximations to the electrochemical environment. This step is therefore a purely comparative measure to see whether a suitable fitting is possible within the expected error range. Naturally, the microkinetic model will provide insight into the performance of individual sites and their ensemble.

### 2.2 DFT‐ΔSCF Method Validation

As the ADF program coupled with the ΔSCF method is rarely employed for the calculation of absolute XPS binding energies, further discussion is needed to assess the accuracy of this approach for subsequent calculations. Regarding the accuracy of the ADF program for molecular core‐level XPS calculations, Yang et al. reported a mean absolute error (MAE) of 0.20 eV for molecular C 1s XPS binding energies of adsorbates bound to a Cu(100) surface. An extrapolation procedure was not needed for this molecular XPS study [23]. Kahk et al. reported an MAE of 0.24 eV for a range of 1s bulk alkali metals, alkaline earth metals, nitrogen, carbon, and oxygen binding energies using the ΔSCF method to extrapolate the binding energy to an infinitely large periodic supercell using the FHI‐aims program [22]. Conversely, the present study employs an extrapolated *cluster* approach, but the accuracy is expected to be comparable to that of the similar extrapolated periodic supercell approach. This supercell approach is partly used to eliminate spurious interactions between periodic images. Favorably, one does not have this issue in cluster systems. One still needs to extrapolate on a cluster basis to reach the infinite cluster basis at a reduced computational effort in comparison to the supercell approach. Such an extrapolation approach is also needed to negate the artificial strain imposed by the finite cluster system. Spin‐polarized calculations for open‐shell systems are also more accessible for increasingly large cluster‐based systems compared to those for large periodic supercells. Due to the local nature of core‐level spectroscopy measurements, the absence of periodicity and suitable expansion of the cluster structure should still capture the relevant interactions.

Although the aforementioned studies provide a good baseline for assessing the accuracy of the ADF program and the ΔSCF method separately, neither has been used together for systems resembling the chemical nature of M–N–Cs. Therefore, it is appropriate to use well‐characterized graphene and nitrogen‐doped graphene (N‐doped) structures to quantify systematic or intrinsic errors in this new approach. Figures of the investigated systems are given as Figures S1–S5. Details of the ΔSCF calculations are given in the Supporting Information section “Computational Details”. The B3LYP functional is chosen based on a study set of small molecule (Figure S9) gas‐phase calculated XPS binding energies that are compared to experiment. Three different DFT functionals are tested in the Supporting Information section “Functional testing XPS small molecules”; the generalized gradient approximation (GGA) Perdew‐Burke‐Ernzerhof (PBE) and hybrid B3LYP and PBE0 functionals. Gas‐phase binding energies are calculated using Equation (8). It is shown in Table S1 that a systematic improvement in binding energy agreement with experiment is found by moving from GGA to hybrid functionals. Ultimately, B3LYP agrees best with experiment across C 1s, N 1s, O 1s, Fe 2p$\frac{3}{2}$, Co 2p$\frac{3}{2}$, and Ni 2p$\frac{3}{2}$ binding energies as shown by the lowest MAEs in Table S2. In some instances, it is shown that binding energies for oxygen‐bound species perform noticeably worse but can be disregarded as they are not relevant in this study. The agreement with metal binding energies is poor across all functionals.

Table 1 presents the calculated extrapolated XPS binding energies for graphene, N‐doped pyridinic, N‐doped pyrrolic, and N‐doped graphitic systems alongside comparisons to experimental XPS binding energies. Two pyrrolic configurations are trialed, namely, a bulk (Figure S4) and an edge (Figure S5) pyrrolic structure. The edge pyrrolic structure agrees within 0.1 eV of experiment, while the bulk structure shows a large deviation of 0.8–0.9 eV $(E_{B}^{\mathrm{VBM}}$‒$E_{B}^{{ɛ}^{\max}})$ from experiment. Relating this observation to thermodynamics, calculations of stoichiometric bulk and edge pyrrolic systems (Figure S15) show that the edge configuration is thermodynamically more favorable than the bulk configuration $\left(\Delta_{\mathrm{bulk}}^{\mathrm{edge}}=-\text{0.14 eV}\right)$. In addition, pyridinic bulk sites formed with a single associated vacancy are thermodynamically unstable in N‐doped graphene, which is problematic as M–N–C structures are typically formed with bulk pyridinic sites [42]. Relevantly, Ibrahim et al. synthesized single‐walled N‐doped nanotubes in a manner that subdued the formation of edge pyridinic sites and promoted the formation of bulk pyridinic sites [40]. Therefore, in this analysis, the binding energy of bulk pyridinic nitrogen in N‐doped nanotubes (397.9 eV) is taken as the pyridinic reference. Also, graphitic sites present a variation in experimental binding energies that are dependent on the position of the site (e.g., bulk or edge). Therefore, a bulk graphitic experimental reference of 400.9 eV is taken to comply with the structure presented in Figure S2 [38]. As shown in Table 1, both extrapolation schemes agree well with experiment, represented by an MAE of $E_{B}^{\mathrm{VBM}}$ = 0.10 eV and $E_{B}^{{ɛ}^{\max}}$ = 0.16 eV, considering both C 1s and N 1s binding energies. Evidently, a previous study showed that ΔSCF calculations of transition‐metal $2p_{\frac{3}{2}}$ absolute binding energies lack sufficient accuracy to be considered reliable for use in this framework [43]. This observation is in agreement with the findings presented on transition metal small molecules in Table S2. Therefore, the N 1s core‐level has been chosen as the critical metric for establishing the quantitative distribution of active sites using the $E_{B}^{\mathrm{VBM}}$ method.

**TABLE 1:** Validation of the extrapolated ΔSCF approach using the VBM and *ɛ* ^max^ methods. Convergence plots for each absolute XPS binding energy are shown in Figure S6. All calculated values are in units of eV.

System		Core level	$E_{B}^{\mathrm{VBM}}$	$E_{B}^{{ɛ}^{\max}}$	$E_{B}^{\mathrm{exp.}}$	$\Delta_{\mathrm{VBM}}^{\mathrm{exp.}}$ ^a^	$\Delta_{{ɛ}_{\max}}^{\mathrm{exp.}}$ ^b^
Graphene	Figure S1	C 1s (C–C)	284.7	284.8	284.6 [37]	−0.1	0.2
N‐doped graphitic	Figure S2	N 1s (N–C)	400.7	400.7	400.9 [38]	−0.2	−0.2
N‐doped graphitic	Figure S2	C 1s (C–N)	285.8	285.7	285.9 [39]	−0.1	−0.2
N‐doped pyridinic	Figure S3	N 1s (N–C)	397.9	397.8	397.9 [40]	0.0	−0.1
Edge N‐doped pyrrolic	Figure S5	N 1s (N–C)	400.1	400.1	400.0 [41]	0.1	0.1

a MAE = 0.10.

b MAE = 0.16.

## 3 Results and Discussion

### 3.1 Pd–N–C Site Analysis

Following the workflow outlined in Scheme 1, an experimental Pd–N–C material is studied to determine the presence and distribution of atomic‐scale active sites. As discussed in the Section 1, the trialed atomic‐scale structures are taken from the literature. Additionally, due to the high percentage of graphitic N found in the experimental reference [24], nitrogen‐enriched Pd–N–C structures (e.g., PdN_4_C_10_ +1N) are investigated. A Pd_4_N_3_C_10_ cluster system is also included to investigate the spectral features and formation energy of a small Pd cluster.

**SCHEME 2:**
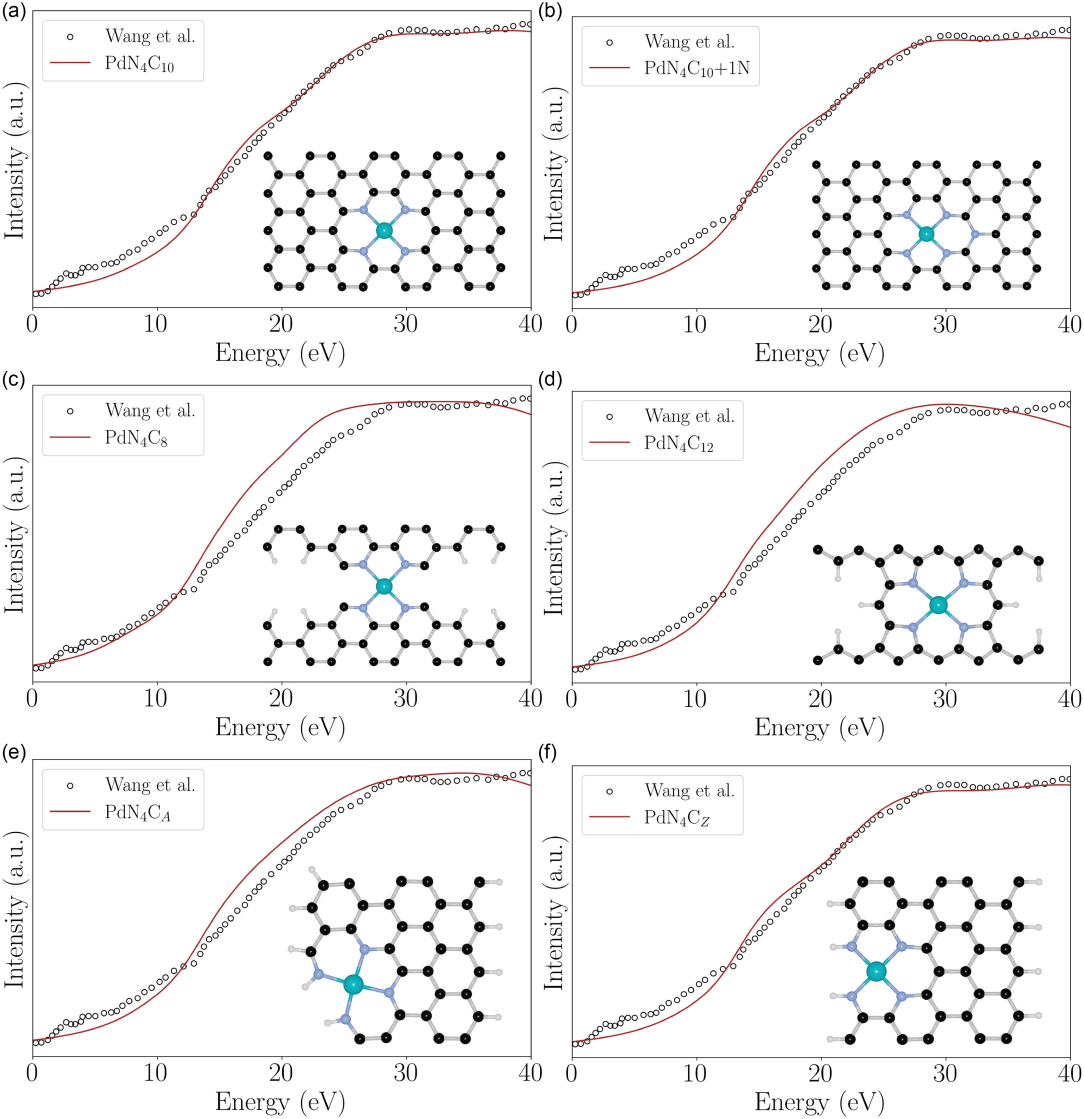
(a–f) Calculated Pd *K*‐edge XANES (red line) with reference to Wang et al. experimental Pd *K*‐edge XANES spectrum (black dots) [24]. (Inset) Atomic‐scale structure; green = Pd, blue = N, black = C, and white = H.

The initial screening step screens structures based on their thermodynamic synthesis energy. A comparison between experimental and computed XANES spectra is a useful sanity measure to check the consistency of the thermodynamics. In Scheme 2, a Pd *K*‐edge spectrum is calculated for each atomic‐scale active site and is given alongside a reference to the experiment [24]. In a previous study, Yang et al. validated the proposed DFT FDMNES finite‐difference procedure for the calculation of XANES spectra for a variety of similar M–N–C systems [44]. Importantly, the finite difference approach eliminates a spurious muffin‐tin potential peak that appears at the onset of the leading edge structure, as shown in Figure S7. Regarding this study, details of the FDMNES calculations are given in the Supporting Information section “Computational Details”. Additionally, the optimized ground state atomic structures provided sufficient spectral agreement with experiment, eliminating the use of structural perturbations or empirical parameter corrections in the FDMNES calculations.

**FIGURE 1:**
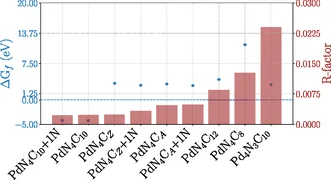
Plotted *R*‐factors (red) of each structure, as shown in Scheme 2 and Figure S8. Also Δ*G*_f_ (blue) for each structure are given. The *R*‐factor value is a measure of agreement between experimental and simulated XANES spectra. Structures are passed to the XPS quantification stage based on Δ*G*_f_ ≤ 0 eV. The Δ*G*_f_ is calculated at the synthesis temperature of 900 °C using *μ*_N_ = −5.67 eV (calculated using an adjustable parameter of *n* = 2.6 in Equation (3)).

As shown in Scheme 2, PdN4C_10_ and PdN4C_10_ + 1N Pd *K*‐edge XANES spectra show a similarly favorable agreement with the experimental reference, reflected also by their near‐equivalent *R*‐factors in Figure 1. A slight underestimation is seen at lower energies, but the agreement at higher energies is excellent. The introduction of an extra nitrogen atom in the first coordination shell increases the formation energy at synthesis and also induces a slight degradation in the *R*‐factor agreement. Previous M–N–C studies are in agreement with these results, showing that the pyridinic site is the most favorable active site to be synthesized. However, the identity of the “other” dominant active site(s) is still ambiguous, especially for Pd–N–C. Here we propose PdN4C_10_ + 1N as the other dominant active site according to the formation energy at synthesis temperature of 900 °C (and chosen nitrogen chemical potential) and XANES analysis. This aligns well with the observation that minimizing the number of nonhexagonal rings reduces structural formation energy; hence, basal C_10_ structures are most likely to form. A detailed DFT structural study of nonhexagonal abundant Fe–N–C structures also found this observation plausible [19]. This does not explain why PdN_4_C_12_ (eight nonhexagonal rings) is much more favorable to form when compared to PdN_4_C_8_ (two nonhexagonal rings). This can be rationalized by the fact that PdN_4_C_12_ contains Stone–Wales defects [45] that redistribute bond strain across five‐ and seven‐membered rings. However, unfavorable formation energies and poor XANES agreement, as shown in Scheme 2, along the rising edge, possibly due to the porosity of bridge PdN_4_C_12_ and PdN_4_C_8_ sites, render these active sites unlikely candidates.

According to synthesis thermodynamics as shown in Figure 1, Pd–N–C edge sites and PdN4C_12_ show approximately equal unfavorable formation energies. The effect of PdN_4_C*_Z_* zigzag‐edge sites compromises the agreement of experiment relative to bulk PdN_4_C_10_ sites, reflected by a slightly larger *R*‐factor. Conversely, for PdN_4_C*_Z_* + 1N, as shown in Figure S8, nitrogen‐doping leads to a noticeable discrepancy in the intensity of low‐energy transitions when compared with the experimental spectrum. This mismatch is reflected in a higher *R*‐factor relative to PdN_4_C*_Z_*, indicating a poorer agreement with experiment. For armchair edge sites, the agreement with experiment is even poorer, especially along the rising edge. The doping with nitrogen (Figure S8) has a negligible impact on spectral features in comparison to its equivalent non‐nitrogen‐doped site. Focusing on Pd_4_N_3_C_10_, the strikingly poor agreement along the rising edge is attributed to a higher number of deep Pd transitions inducing noticeably stronger intensities along this range. Interestingly, the following trend in thermodynamic Δ*G*_f_ energies can be observed; C_10_ < edge/zigzag < C_12_ < C_8_ sites. If the same grouping is used for the spectroscopic *R*‐factor values, a similar trend also arises, hence showing consistency between both thermodynamic and spectroscopic measures. Ultimately, bulk C_10_ sites are shown as clearly plausible sites to be present in the distribution. One obvious outlier here is the Pd_4_N_3_C_10_ structure, but it is clearly not favorable to form nor agreeable with XANES experiment; hence, it can be disregarded.

**FIGURE 2:**
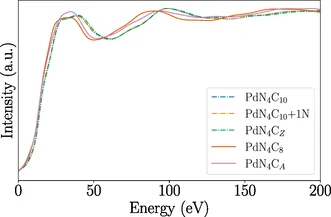
Calculated EXAFS spectra of selected Pd–N structures. PdN_4_C_10_, PdN_4_C_10_ + 1N, and PdN_4_C*_Z_* (dotted lines) are indistinguishable over the EXAFS range. PdN_4_C_A_ and PdN_4_C_8_ (solid lines) are shown as reference structures that differ considerably from dotted structures over the sampled EXAFS range.

Extended X‐ray absorption fine structure (EXAFS) spectra encode a wealth of geometric data, including coordination numbers and bond distances that could further strengthen the reliability of the XANES screening procedure. While experimental EXAFS data for this literature sample is unavailable, computed spectra for several candidates are presented nevertheless in Figure 2. The analysis reveals that the spectra for PdN_4_C_10_, PdN_4_C_10_ + 1N, and PdN_4_C*_Z_* are essentially indistinguishable, suggesting that EXAFS alone cannot differentiate between these specific configurations. These three structures share an identical coordination number in the Pd–N shell and maintain similar average Pd─N bond distances between 1.924 and 1.957 Å (Table 2). In contrast, the PdN_4_C*_A_* and PdN_4_C_8_ structures exhibit considerable spectral variation primarily due to elongated Pd─N bonds. Ultimately, due to these spectral similarities, XANES/EXAFS measurements lack the resolution required to definitively distinguish among PdN_4_C_10_, PdN_4_C_10_ + 1N, and PdN_4_C*_Z_* sites.

**TABLE 2:** DFT calculated bond distances.

	PdN_4_C_10_	PdN_4_C_10 _+ 1N	PdN_4_C*_Z_*	PdN_4_C_A_	PdN_4_C_8_
Pd‐N1, Å	1.954	1.957 (+1N side)	1.947	2.003	2.046
Pd‐N2, Å	1.954	1.957 (+1N side)	1.947	1.960 (bound to H)	2.046
Pd‐N3, Å	1.954	1.945	1.924 (bound to H)	2.008	2.046
Pd‐N4, Å	1.954	1.945	1.924 (bound to H)	1.995 (bound to H)	2.046

**FIGURE 3:**
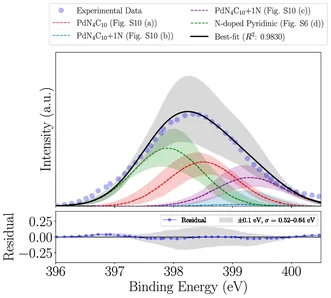
(Top panel) (blue dots) Pyridinic N 1s peak obtained by Shirley background subtraction and peak deconvolution of the raw experimental spectrum reported in Ref. [24]. (Black curve) The best‐fit summation of each individual active site Gaussian peak is optimized until a satisfactory agreement with experiment is found. The shaded areas are the deviation from the best‐fit after applying a systematic error of ±0.1 eV to all peak binding energies and a ±10% perturbation to the peak width. The gray shaded area is the result of combining the errors of individual peaks. The figure label in parentheses shows where the absolute XPS value is calculated using the extrapolated ΔSCF procedure. (Bottom panel) Residuals of the best‐fit (circles) and the errors (gray) of the summed peak.

Therefore, based on favorable thermodynamic formation energies (Δ*G*_f _ ≤  0 eV), we proceed with PdN_4_C_10_ and PdN_4_C_10_ + 1N to the distribution quantification analysis shown in Figure 3. It is noted that *μ*_N_ = −5.67 eV is used to calculate Δ*G*_f_ (corresponding to an adjustable parameter of *n* = 2.6 in Equation (3)) in Figure 1 and Table 3. Under reasonable synthesis conditions (*n* > 2), PdN_4_C_10_ and PdN_4_C_10_ + 1N remain the only thermodynamically favorable structures to form. Furthermore, this analysis does not conclusively identify these as the only sites within the experimental sample because (1) only a finite number of sites can be evaluated and (2) errors in the model’s data or underlying assumptions may lead to the erroneous exclusion of valid sites. Moreover, if the spectroscopic *R*‐factor value is used as the sole identification parameter without regard for thermodynamic stability, PdN_4_C*_Z_* is a viable candidate to be included in the calculated distribution. The sensitivity of the distribution to the inclusion of PdN_4_C*_Z_* is therefore also assessed in the quantification study.

**TABLE 3:** Tabulated Δ*G*_f_ and *R*‐factors of all active sites from Figure 1. Active sites with a Δ*G*_f_ ≤ 0 eV proceed to the distribution analysis, as shown in Figure 3.

Structure	Δ*G*_f_, eV	*R*‐factor
PdN_4_C_10_	−4.23	0.00231
PdN_4_C_10 _+ 1N	−4.20	0.00257
PdN_4_C*_Z_*	3.45	0.00260
PdN_4_C_*Z* _+ 1N	3.03	0.00404
PdN_4_C*_A_*	3.30	0.00585
PdN_4_C*_A _* + 1N	2.97	0.00587
PdN_4_C_12_	4.20	0.00959
PdN_4_C_8_	11.38	0.01264
Pd_4_N_3_C_10_	3.15	0.02681

While X‐ray absorption spectra and thermodynamic formation analysis serve as a qualitative screening tool for identifying plausible structures, XANES lacks the ability to quantitatively resolve the distribution of active sites within a material. To address this limitation, we introduce a quantifiable approach that employs the extrapolated ΔSCF method to calculate the absolute N 1s binding energies of each site that passes the initial screening. Details of the ΔSCF calculation setup are given in the Supporting Information section “Computational Details”, alongside XPS‐extrapolated ΔSCF convergence plots in Figure S10. Note that the VBM approach is used exclusively owing to its lower MAE values in comparison to the *ɛ*_max_ approach (Table 1).

As discussed in Section 2, an SLSQP optimization of the residual sum of squares is used to find the relative intensities. The ratio of each area is then taken as the distribution that best represents the experimental sample of interest. In other words, this is taken as the best‐fit estimate within the assumptions imposed by the model. For the example Pd–N–C system, the distribution of each active site is shown in Figure 3, where the experimentally background‐corrected deconvoluted N 1s XPS pyridinic peak is taken as the critical peak for comparison of experiment with calculation. The authors achieved this by digitizing the raw experimental N 1s spectrum and applying a Shirley background correction for ease of comparison with the computed spectrum [46]. The full width at half maximum (FWHM) is refitted from the original raw spectrum as a result. It is assumed that all active sites that contribute to the distribution are found within the pyridinic peak, which is reasonable based on the prominence of pyridinic sites acting as metal active site anchors in the literature [1, 47]. Moreover, previous experimental XPS studies have speculated that the N–Pd and pyridinic peaks are contained within the same major peak. Yet, the exact N–Pd peak energy remains speculative [24, 48]. A previous study showed that the M–N–C N–metal peak is typically found at the higher energy side of the pyridinic peak for a variety of other SACs [47]. The theoretical N–Pd deconvolution presented here aligns well with experimental observations, thus showing consistency between experimental and theoretical characterization.

In Table 4, the calculated distribution (i.e., % of summed peak) is shown alongside the calculated absolute binding energies ($E_{B}^{\mathrm{VBM}}$), and the difference between the pyridinic experimental reference and calculated value ($\Delta_{\exp.}^{\mathrm{pyrid}.}$). For completeness, the calculated PdN_4_C_10_ + 1N graphitic binding energy (Figure S10d) is presented and is shown to be contained within the experimental graphitic peak ($\Delta_{\exp.}^{\mathrm{graph}.}$). According to the XPS analysis, nitrogen‐doping of bulk PdN_4_C_10_ + 1N in the first coordination shell shows good agreement with experiment, as the two calculated pyridinic environments match closely with the corresponding experimental N 1s peaks. It is noted that if a structure contains more than one unique N–Pd peak (i.e., PdN_4_C_10_ + 1N), their respective distributions are summed to represent that structure in the final best‐fit distribution. Interestingly, metal free N‐doped pyridinic sites are inactive in acidic media [49]. Thus, after removing the pyridinic contribution and renormalizing the two Pd‐active‐site configurations (PdN_4_C_10_ at 33% and PdN_4_C_10_ + 1N at (22 + 1)% from Table 4), the final distribution is PdN_4_C_10_: 59% and PdN_4_C_10_ + 1N: 41%. This is in excellent agreement with the experimental spectrum (*R* ^2^ = 0.9830). *σ* was determined to be 0.58 eV from the FWHM of the Shirley corrected experimental Pd–N peak [24]. A previous study of SACs showed that a Boltzmann population analysis is a viable approach for determining distributions using thermodynamics exclusively [50]. Hence, the weighted Boltzmann population for this study is calculated using Equation (10) at a synthesis temperature of 900^∘^ C and a *μ*_N_ = −5.67 eV. This corresponds to a parameter choice of *n* = 2.6 in Equation (3), which lies above the threshold of *n* = 2 and is typical of high‐temperature pyrolytic synthesis of SACs. The choice of *n* is based on finding the best fit possible to the spectroscopically determined distribution. Using the Gibbs free energies in Table 3, a Boltzmann distribution of 58% and 42% is calculated for PdN_4_C_10_ and PdN_4_C_10_ + 1N, respectively. A 5% error in *μ*_N_ sees a near‐unity contribution from PdN_4_C_10_; hence, one must be aware that this calculated distribution is a result of the chosen *μ*_N_, and that *μ*_N_ is especially sensitive to the choice of *n*.

**TABLE 4:** Tabulated XPS binding energies ($E_{B}^{\mathrm{VBM}}$) from Figures 3 and S11 alongside the difference of each calculated pyridinic and graphitic peak from experiment where applicable ($\Delta_{\exp.}^{\mathrm{pyrin}.}$, $\Delta_{\exp.}^{\mathrm{graph}.}$).

System	Label	$E_{B}^{\mathrm{VBM}}$, eV	$\Delta_{\mathrm{exp.}}^{\mathrm{pyrid.}}$, eV	$\Delta_{\mathrm{exp.}}^{\mathrm{graph.}}$, eV	% of summed peak
PdN_4_C_10_	Figure S10a	398.5	−0.3	—	33
PdN_4_C_10_ + 1N	Figure S10b	399.1	−0.9	—	1
PdN_4_C_10_ + 1N	Figure S10c	399.3	−1.1	—	22
PdN_4_C_10_ + 1N	Figure S10d	401.6	—	−0.8	—
N‐doped pyridinic	Figure S6d	397.9	0.6	—	44

*Note* : The “% of summed peak” column indicates the percentage contribution of each individual Gaussian to the total peak. Labels correspond to their respective extrapolated ΔSCF convergence plot.

For brevity, the remaining analyses therefore trial only Gaussian line shapes unless otherwise stated. A sensitivity analysis is performed to assess potential errors in the reported distribution in Table S3. Realistic perturbations in the binding energy of all peaks (systematic and independent) and the assigned peak width are given. Upon application of reasonable physical perturbations to the above parameters, the reported error in the distribution spans from 67% to 51% and 49%–33% for PdN_4_C_10_ and PdN_4_C_10 _+ 1N, respectively. The largest divergence from the best‐fit distribution is observed by perturbing the peak width by +10%. The physical basis for assigning the chosen peak width is based on the peak width of the experimental N 1s spectrum; hence, any error would be a consequence of the experimental fitting. A combination of plausible perturbations is also given in Table S3. A systematic perturbation to all peaks of +0.1 eV and −10% peak width incurs the worst‐case scenario shifting the distribution to 47% and 53% for PdN_4_C_10_ and PdN_4_C_10_ + 1N, respectively. The visual effect of systematic perturbations on binding energies and peak widths is shown by the shaded areas in Figure 3. Additionally, the pyridinic PdN_4_C_10_ and PdN_4_C_10_ + 1N peaks can be particularly challenging to resolve if moved independently; PdN_4_C_10_ by +0.1 eV and PdN_4_C_10_ + 1N by −0.1 eV. This causes the separation between the peaks to decrease from 0.6 to 0.4 eV, causing a total redistribution of each site’s assigned area; 17% and 83% for PdN_4_C_10_ and PdN_4_C_10_ + 1N, respectively. Therefore, if independent peak perturbations are pertinent, this result indicates that 0.6 eV is the resolution limit at an FWHM of 1.37 eV. Ultimately, any errors are predicted to be systematic due to the chemical similarity of sites, thus avoiding independent overlap of peaks and loss of resolution. A Voigt line shape fitted to the binding energies in Table 1 is also shown in Figure S11. The total FWHM was fixed to that of the Gaussian fit (2$\sqrt{2\ln2}\sigma$ ≈ 1.37 eV), with the Lorentzian width of the Voigt profile fitted and its Gaussian width adjusted accordingly, so that any differences between the two fits arise solely from the line shape. The summed Voigt distribution (PdN_4_C_10_: 62% and PdN_4_C_10_ + 1N: 38%) remains similar to the Gaussian fit, with a minor redistribution of weight between both PdN_4_C_10_ + 1N sites being the only notable change. Indeed, additional errors may arise due to imprecise XPS line fitting and/or background corrections; therefore, one must be aware that unanticipated large errors may cause significant shifting of the distribution. It is difficult to quantify the effect of all such errors, especially if the experimental spectra are taken from literature.

Finally, Table 3 shows nearly equivalent *R*‐factor values for PdN_4_C_10_, PdN_4_C_10_ + 1N, and PdN_4_C*_Z_*. It is therefore of interest to include PdN_4_C_Z_ to determine its effect on the resulting distribution. A distribution of 44%, 14%, and 42% is calculated for PdN_4_C_10_, PdN_4_C_10_ + 1N, and PdN_4_C*_Z_* respectively. While PdN_4_C_10_ remains dominant, the contribution from PdN_4_C_10 _+ 1N is significantly reduced. However, in light of the unfavorable Gibbs free formation energy for PdN_4_C*_Z_*, this specific distribution can be disregarded from further analysis.

### 3.2 Microkinetic Distribution Analysis

A microkinetic model will allow one to check if the calculated distribution remains compatible between core‐level spectroscopy measurements and electrocatalytic operation within plausible uncertainty due to DFT functional error. Furthermore, analyzing the reaction rates through a microkinetic model provides physical insight into the rate‐controlling steps and intermediate coverages that govern the overall mechanism. Previous ORR studies have identified individual steps as rate‐determining [51, 52], yet a single step is rarely dominant over the working potential range. Therefore, it is applicable to define the degree of rate control (DRC) [53], which quantifies the sensitivity of the reaction rate *r* toward reaction step *i*

(11) $$X_{\mathrm{RC},i}={\left(\frac{\partial \;\ln\;r}{\partial \;\ln\;k_{i}}\right)}_{k_{j\neq i},K_{i}}$$

where *k*_*i*_ is the forward rate constant and *K*_*i*_ is the equilibrium constant of step *i*. Steps with a positive DRC will increase the net rate if the transition state is stabilized; conversely, steps with a negative rate control step will increase the net rate if destabilized. Details of the mean‐field microkinetic model are given in the Supporting Information section “Microkinetic Model”.

**FIGURE 4:**
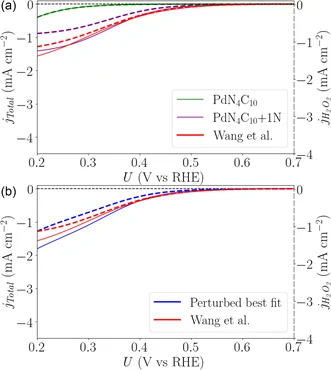
Comparison of total calculated (solid line measured using the left *y*‐axis) and partial H_2_O_2_ current (dashed line measured using the right *y*‐axis); (a) of the perturbed individual active site. (b) The active site ensemble after applying the computed distribution with reference to Wang et al. experiment [24]. All polarization curves are calculated at 1600 rpm.

As shown in Figure S12b, the agreement with the rotating ring disk electrode (RRDE) experiment is poor for the summed unperturbed PdN_4_C_10_ and PdN_4_C_10 _+ 1N systems, possibly due to approximations in the electrochemical environment and/or DFT functional error. While microkinetic modeling serves as a useful framework for identifying qualitative trends, its quantitative predictive power is often constrained by the exponential sensitivity of rate constants to uncertainties in computed electronic structure energies [36]. Therefore perturbations in the energies are permitted to improve fit with experiment. In the perturbed individual systems (Figure 4a), ΔG(OOH*) perturbations within DFT error of 0.07 and −0.14 eV are applied to PdN_4_C_10_ and PdN_4_C_10_ + 1N, respectively. ΔG(OOH*) is chosen as it is the key intermediate that affects selectivity between the two‐ and four‐electron routes. A satisfactory perturbed fit is observed in Figure 4b, with the largest deviations occurring at around 0.30  to 0.40 V. One can argue that, within the perturbed DFT error range, the distribution of active sites remains compatible between core‐level spectroscopy and electrocatalytic measurements within the assumed energetic perturbations.

**FIGURE 5:**
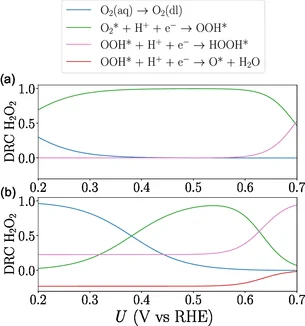
Degree of rate control (DRC) of H_2_O_2_ production for (a) PdN_4_C_10_ and (b) PdN_4_C_10 _+ 1N active sites. Only degrees of rate control larger than 0.01 have been shown for clarity.

According to the H_2_O_2_ production DRC analysis for both active sites (a) and (b) in Figure 5, the O_2_*  →  OOH* step is the dominant activity onset controlling step for both sites’ RRDE polarization curve in Figure 4a. Once the Fermi level of the electrons in the electrode is tuned to the appropriate energy, this reaction step becomes downhill in energy, allowing a total net current flow. As shown by an earlier onset potential in Figure 4a, PdN_4_C_10 _+ 1N reaches a more favorable energetic state for current flow earlier than PdN_4_C_10_. As the proton coupled electron transfer barriers at equilibrium for all steps of each active site are assumed to be 0.26 eV, thermodynamics will dictate the activity and selectivity of the active site. For the PdN_4_C_10 _+ 1N site in Figure 5b, the mass transport step O_2_(aq)  →  O_2_(dl) begins to exert a measurable influence on the total reaction rate at ≈0.45 V. This onset of mass transport sensitivity precedes its near unity DRC sensitivity, as evidenced by the mass transport limited trend over the same voltage range in Figure 4a. The DRC of PdN_4_C_10_ shows that the total net current becomes sensitive to mass transport limitations at a higher overpotential of ≈0.30 V, yet still remains insignificant in comparison to the O_2_*   →  OOH* step; hence, mass transport limitations are not observed in Figure 4a. A high degree of mixing from the PdN_4_C_10_ site dampens any mass transport limitations in the ensemble structure (Figure 4b) that would be emergent due to the PdN_4_C_10_+1N site. Compared to the highly active cobalt‐based H_2_O_2_ sites, both Pd sites exhibit inferior activity. This performance gap is directly attributable to the weak binding of intermediates, which results in suboptimal Sabatier behavior, therefore explaining the poor experimental activity. Reflecting on the active sites’ sensitivity toward perturbations in ΔG(OOH*) (Figure S12a), PdN_4_C_10_ exhibits a higher nonperturbed activity, while PdN_4_C_10 _+ 1N is significantly more active, especially at higher overpotentials in comparison to their perturbed structures. An overbinding of oxygen species due to errors in the DFT functional justifies such a perturbation in the ΔG(OOH*) energy in order to weaken the interaction of species with the metal site.

Examining the selectivity of each active site, PdN_4_C_10_ shows 100% selectivity toward the 2e^−^ pathway over the working potential range, as shown in Figures 4a and S13. Conversely, PdN_4_C_10 _+ 1N shows competing 2e^−^ (63%) and 4e^−^ (37%) selectivities, therefore potentially explaining the active site origin of competing pathways in the experimental sample. Over the experimental working range (Figure4b), the H_2_O_2_ selectivity is dynamic—which is not observed in the constant selectivity single‐site regime (Figure S13). Transitioning to the summed site regime in Figure 4b, one captures dynamical changes in H_2_O_2_ selectivity, although quantitative parity remains elusive. The H_2_O_2_ DRC analysis in Figure 5 shows that the OOH*  →  O* step for PdN_4_C_10 _+ 1N partially inhibits the H_2_O_2_ rate, accounting for the reduction in 2e^−^ selectivity. This effect is not present for PdN_4_C_10,_ as the H_2_O_2_ rate is solely sensitive to the OOH* state. PdN_4_C_10 _+ 1N shows stronger adsorption binding energies of intermediates as compared to PdN_4_C_10_, aiding the irreversible scission of the *O─OH bond and therefore reducing H_2_O_2_ selectivity [54]. Surface coverages in Figure S14 show that the empty site is dominant over the entire working range at the steady state, which is reasonable considering that intermediate species bind weakly to the active site. Rather than following a single rate‐limiting step, this analysis shows that the total rate is sensitive to multiple steps (typically the O_2_* → OOH* or mass transport steps) over the working potential range, which is critical for capturing the dynamic activity and selectivity trends that align more closely with experimental observations.

## 4 Framework Limitations

While this combined spectroscopic and microkinetic framework demonstrates a successful approach for the Pd–N–C material studied, there are cases where additional caution is warranted. One of the key assumptions of this paper’s active site distribution analysis is that perturbations of the surface are only felt within a radius of a few angstroms. This electronic screening or “nearsightedness” essentially allows one to view active sites as independent of each other, permitting simple summation [55, 56]. This can be rationalized by the fact that, for a fixed chemical potential, the electron density n(*r*) at a given point depends significantly on the effective external potential only in its immediate vicinity. Critically, locality supports the assumptions of Brønsted–Evans–Polanyi (BEP) [57] and Sabatier [58] relations for use in relating local structure to the energetics of catalysis. Conversely, cooperative effects sometimes cannot be avoided and can unpredictably influence reaction energetics, undermining the use of summative independent kinetic models. Indeed, interacting active sites could activate multiple reactants simultaneously through adsorbate–adsorbate interactions or kinetic coupling via desorption–diffusion–adsorption [59], thus enabling unpredictably high activities and/or selectivities. It is also assumed that the active sites remain static over the electrocatalytic operating range. In fact, these active sites can evolve; therefore, operando XPS and X‐ray absorption spectroscopy (XAS) could be used to monitor structural changes under operation. From the current computational perspective, calculating operando XPS and XAS spectra from first‐principles is challenging; hence, machine learning techniques could be leveraged to accelerate exploration [60].

A caveat that cannot be resolved with this framework is that one needs to define a critical XPS binding energy that allows sufficient resolution of unique active sites (e.g., the pyridinic peak in SACs). Additionally, if the system consists solely of transition metal atoms, the ΔSCF method lacks sufficient accuracy for calculating absolute XPS binding energies [43]. If a consistent correction that shifts calculated XPS binding energies to experiment were found, this avenue for comparison could become viable. Moreover, the FDMNES program is validated for computing accurate *K*‐edge spectra for a variety of metals, but this edge may not be accessible due to the constraints in the beam line energy. *L*‐edge spectra are often more experimentally accessible; however, the accurate calculation of *L*‐edge spectra is hindered, for example, by an inability to reproduce excitonic states that give rise to multiplet features due to the ground state nature of the ΔSCF method. Wavefunction‐based methods could potentially reproduce such features, but as of now, such methods are extremely computationally demanding.

Regarding XANES spectral fitting, inherent limitations exist due to the sheer number of fitting parameters that exist, which can lead to nonunique solutions. Although unperturbed XANES calculations are preferred, many systems require geometric or empirical adjustments to achieve agreement with experiment. FDMNES partially addresses this by permitting physically constrained structural perturbations. Machine learning‐assisted parameter exploration complemented by hybrid experimental–theoretical constraints has been shown to be effective in overcoming such limitations [60, 61, 62] but has not been widely implemented as of yet. The ΔSCF XPS quantification approach also relies significantly on the quality of the fitting procedure. The sensitivity of the calculated distribution to the appropriate fitting parameters is discussed in detail in the text. A DFT spin density analysis aided by experimental magnetic susceptibility measurements could also be a useful support step in the framework [13].

## 5 Conclusion

In summary, we present a framework that identifies active sites and quantifies their respective distributions within an experimental Pd–N–C sample taken from literature using a validated coupled experimental and computational characterization approach. A validation study of small molecules followed by nitrogen‐doped graphene supports the use of the ΔSCF method for calculating absolute XPS values using the VBM approach (MAE = 0.10 eV). The initial thermodynamic screening step of the framework reveals that the PdN_4_C_10_ and PdN_4_C_10 _+ 1N configurations retain favorable synthesis energies under experimental synthesis conditions. Complemented by XANES, both sites exhibit spectra in excellent agreement with the background corrected experimental spectrum, indicating their likely presence within the sample. Relative site intensities were obtained by an SLSQP fitting of the calculated binding energies to the pyridinic experimental N 1s peak after applying a background correction of the published spectrum in this work. An active site distribution of PdN_4_C_10_: 59 and PdN_4_C_10_ + 1N: 41% reveals that basal plane bulk sites dominate the experimental Pd–N–C sample. The above distribution is the best‐fit value according to the model’s assumptions, and other sites may exist outside the bounds of this model. A sensitivity analysis assigns a range of 67%–51% and 49%–33% to PdN_4_C_10_ and PdN_4_C_10_+1N, respectively, based on realistic systematic errors in the calculated binding energies and assigned peak width. A Boltzmann analysis with the nitrogen chemical potential *μ*_N_ as the sole fitted parameter reproduces the spectroscopically deduced site populations, supporting their assignment. Furthermore, an ORR microkinetic model confirms that the spectroscopically deduced distribution is compatible with the calculated polarization curves within the assumed energetic perturbation and error of the DFT functional used. A microkinetic analysis of individual sites shows that the majority of the perturbed ensemble activity originates from the PdN_4_C_10 _+ 1N site, especially at higher overpotentials. Mass transport limitations are alleviated by the PdN_4_C_10_ site. A decrease in 2e^−^ selectivity is induced by nitrogen‐doping of the basal structure, which is necessary to fit the experiment. Interestingly, dynamical changes in selectivity that are characteristic of the experiment can only be reproduced in the ensemble site regime as opposed to the single‐site regime. This work provides a computationally supported framework for characterizing and determining the active site distributions of SACs in an experimental sample—allowing one to form a more physically accurate representation of the active site structure.

## Funding

This work was supported by the Novo Nordisk Fonden (NNF22OC0078009), Thomas B. Thriges Foundation (5127‐2502), Otto Mønsteds Fond (24‐70‐2548), European Cooperation in Science and Technology (CA21101), and Danmarks Frie Forskningsfond (3105‐00203A).

## Conflicts of Interest

The authors declare no conflicts of interest.

## Supporting information

Additional information on calculation setup, XPS, XANES, and microkinetic spectra. Additional details of the microkinetic model are also given.

## Data Availability

Input files for XANES/XPS calculations and optimized XYZ geometries will be available at DTU Data, DOI: 10.11583/DTU.30416629, upon publication.
