# Supplementary material for: An Integrated Spectroscopy–Microkinetic Approach to Identifying Active‐Site Distributions in Palladium Single‐Atom Catalysts
Source: ChemSusChem. 2026 Sep 7;19(17):e71026. doi: 10.1002/cssc.71026 (PMC13550710; doi:10.1002/cssc.71026)
Supplement: Supplementary file 1 [file CSSC-19-e71026-s001.pdf]

SUPPORTING INFORMATION - An Integrated Spectroscopy–Microkinetic Approach to Identifying Active-Site Distributions in Palladium Single-Atom Catalysts

Ronan Gleeson,[†] Alessandro Fortunelli,[‡] Mathias Meidahl Hommelgaard,[†] Mauro Stener,[¶] Yang Hu,[†] and Heine Anton Hansen[*,†]

†*Department of Energy Conversion and Storage, Technical University of Denmark, 2800 Kongens Lyngby, Denmark.*

‡*CNR-ICCOM, Consiglio Nazionale delle Ricerche, 56124 Pisa, Italy.*

¶*Dipartimento di Scienze Chimiche e Farmaceutiche, University of Trieste, 34127, Trieste, Italy.*

E-mail: heih@dtu.dk

Contents

N-Doped Systems Validation **3**

Pd-N-C XANES Muffin-Tin **7**

Pd-N-C Pd *K*-edge XANES **8**

Functional testing XPS small molecules **9**

Pd-N-C Extrapolated ΔSCF Convergence Plots **14**

Sensitivity Analysis **15**

Additional Microkinetic Modelling Curves **17**

Computational Details **18**

Bulk and Edge N-doped System Thermodynamics **19**

Adsorption Thermodynamics **20**

Microkinetic Model **23**

N-Doped Systems Validation

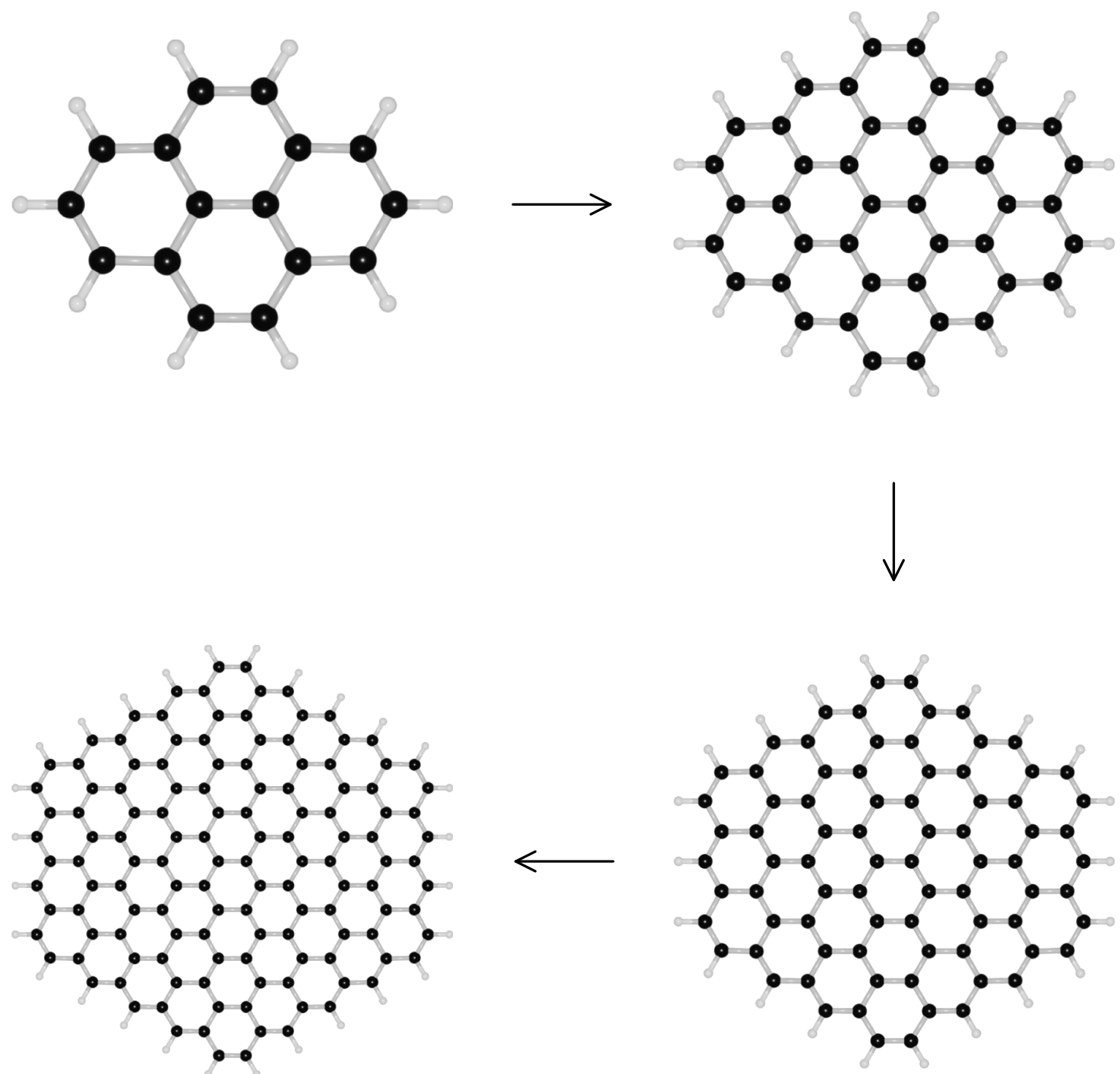

Figure S1: Extended graphene systems

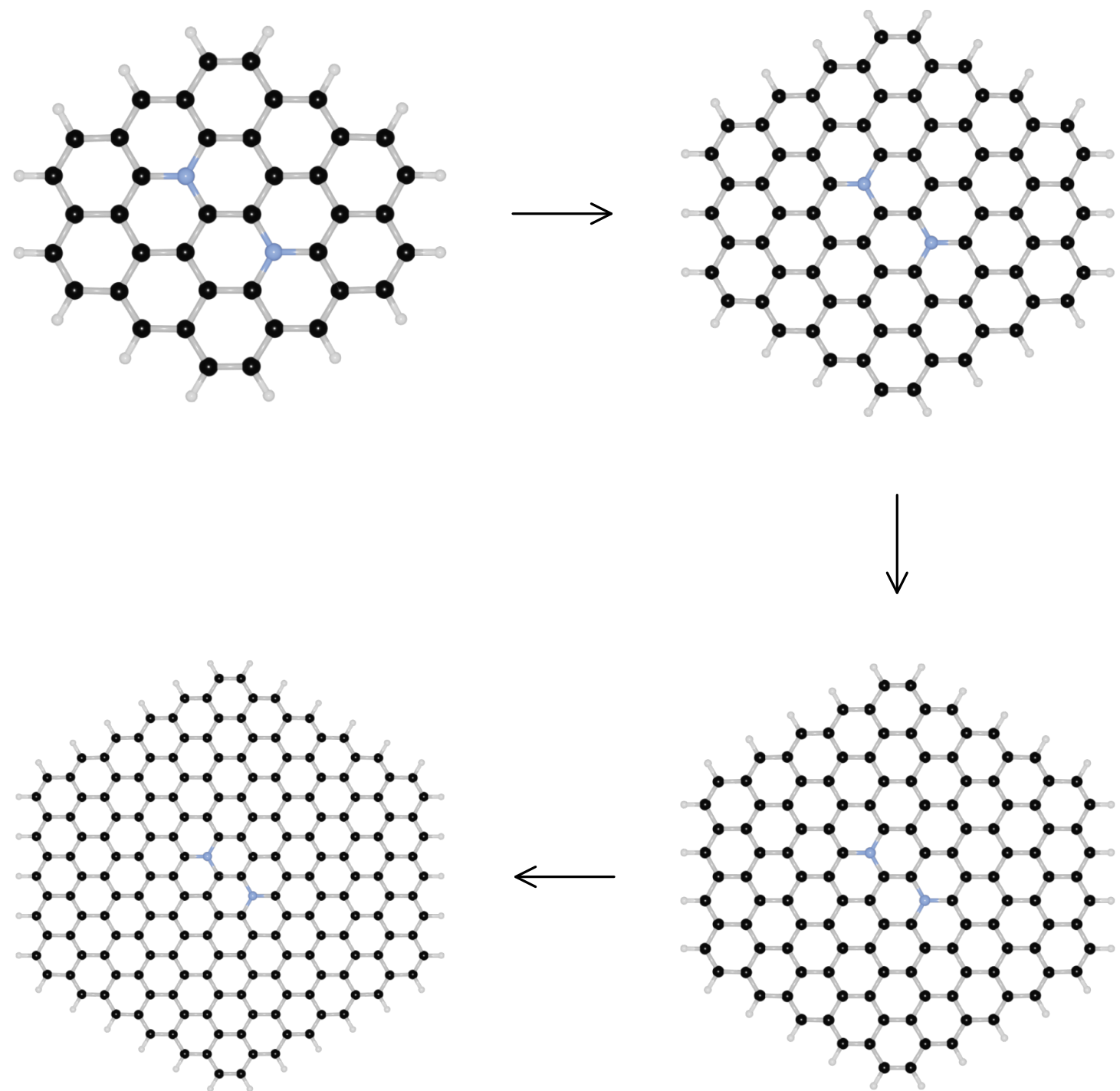

Figure S2: Extended N-doped graphitic systems

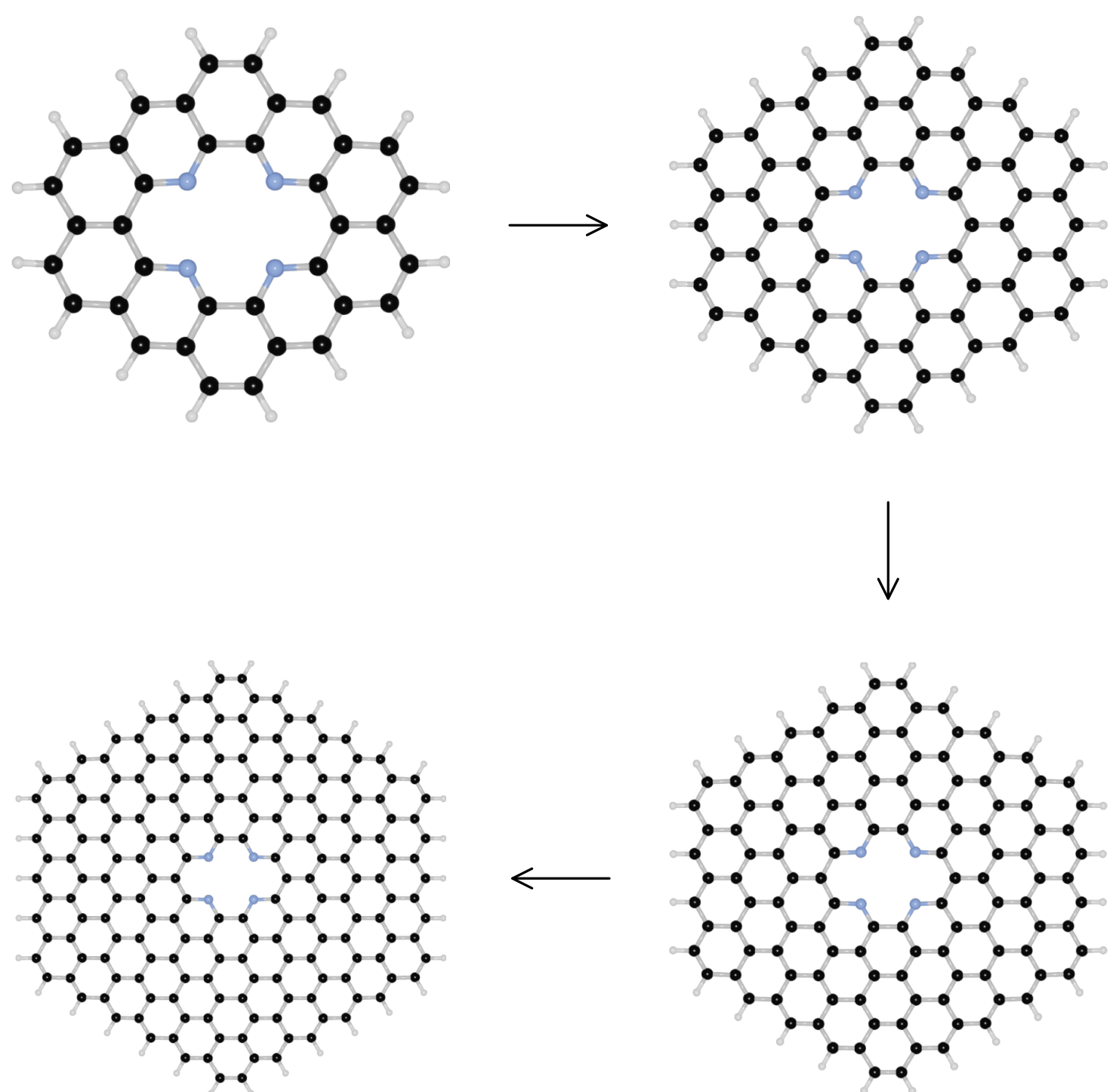

Figure S3: Extended N-doped pyridinic systems

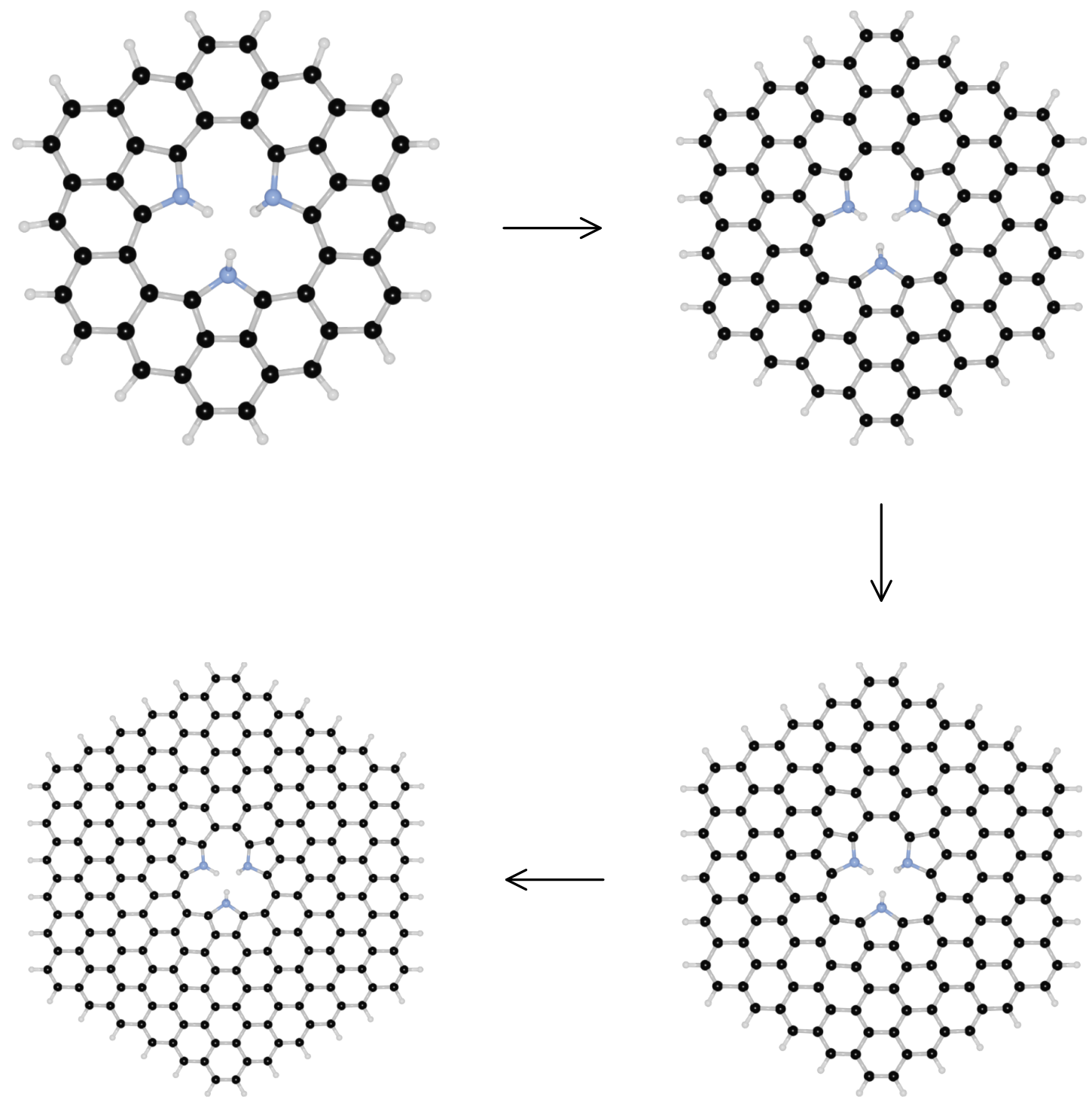

Figure S4: Extended bulk N-doped pyrrolic configuration 1

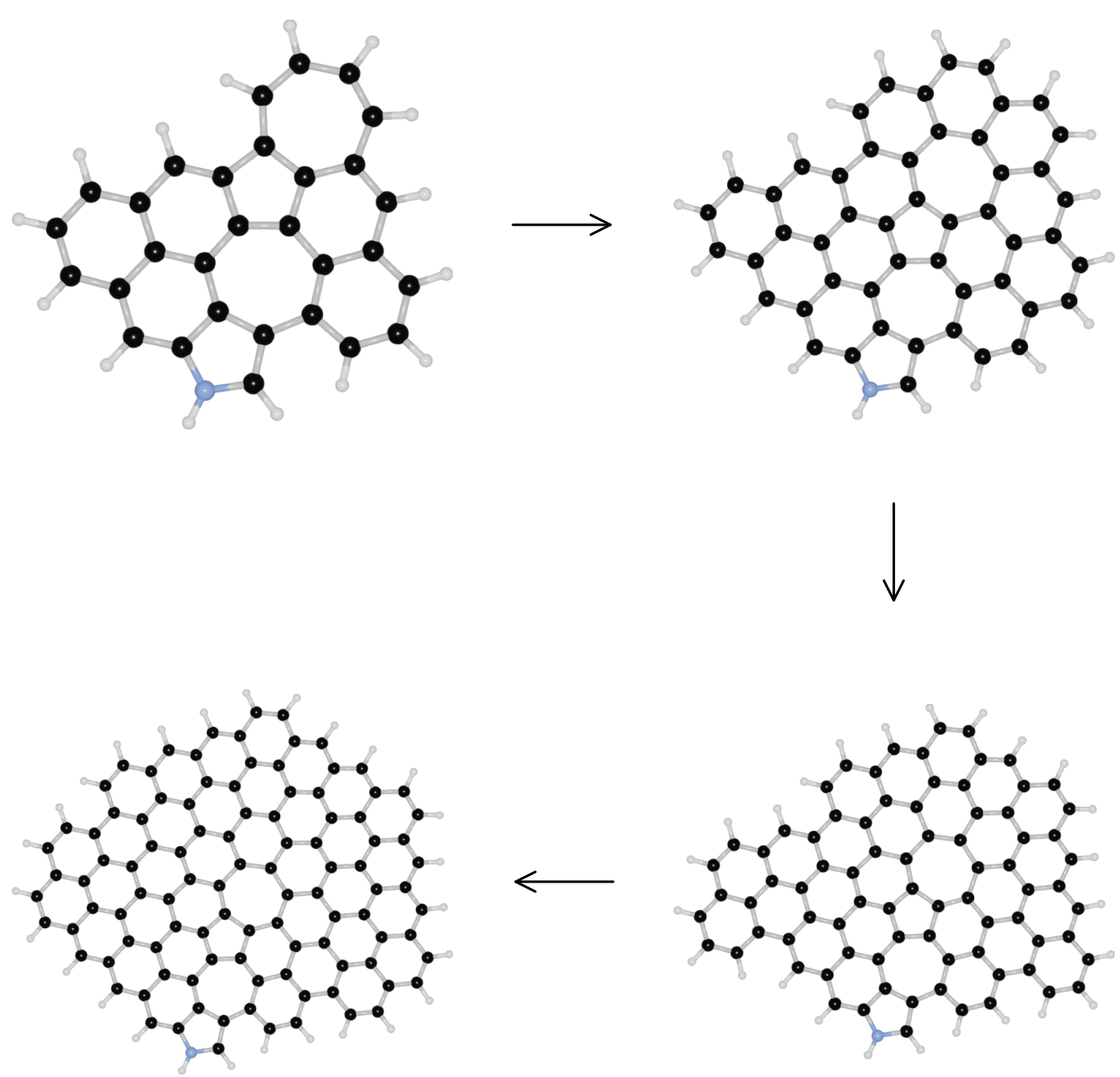

Figure S5: Extended edge N-doped pyrrolic configuration 2

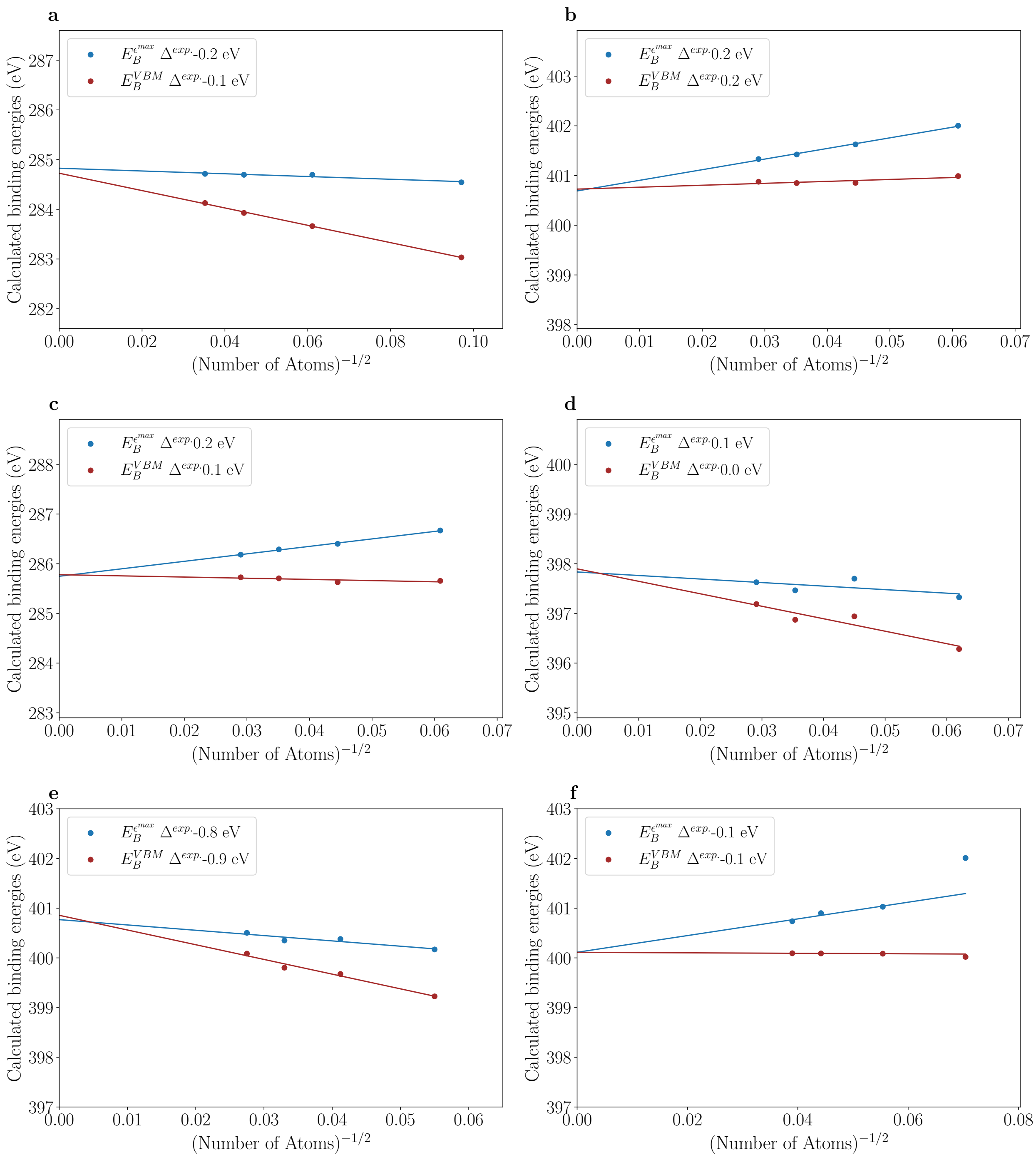

Figure S6: Calculated binding energies for the VBM and ϵ^{max} methods to the limit of the (Number of Atoms)$^{-1/2}$ where $\Delta^{exp.} = E_B^{exp.}$ - E_B. a) C1s graphene (Fig. S1), b) N1s N-doped graphitic (Fig. S2), c) C1s N-doped graphitic (Fig. S2), d) N1s N-doped pyridinic (Fig. S3), e) N1s N-doped pyrrolic configuration 1 (Fig. S4), f) N1s N-doped pyrrolic configuration 2 (Fig. S5). In Fig. S5, the extrapolated ϵ^{max} line is not fitted through point (0.07, 402.2) as non-physical and non-linear effects can influence smaller clusters[1]

Pd-N-C XANES Muffin-Tin

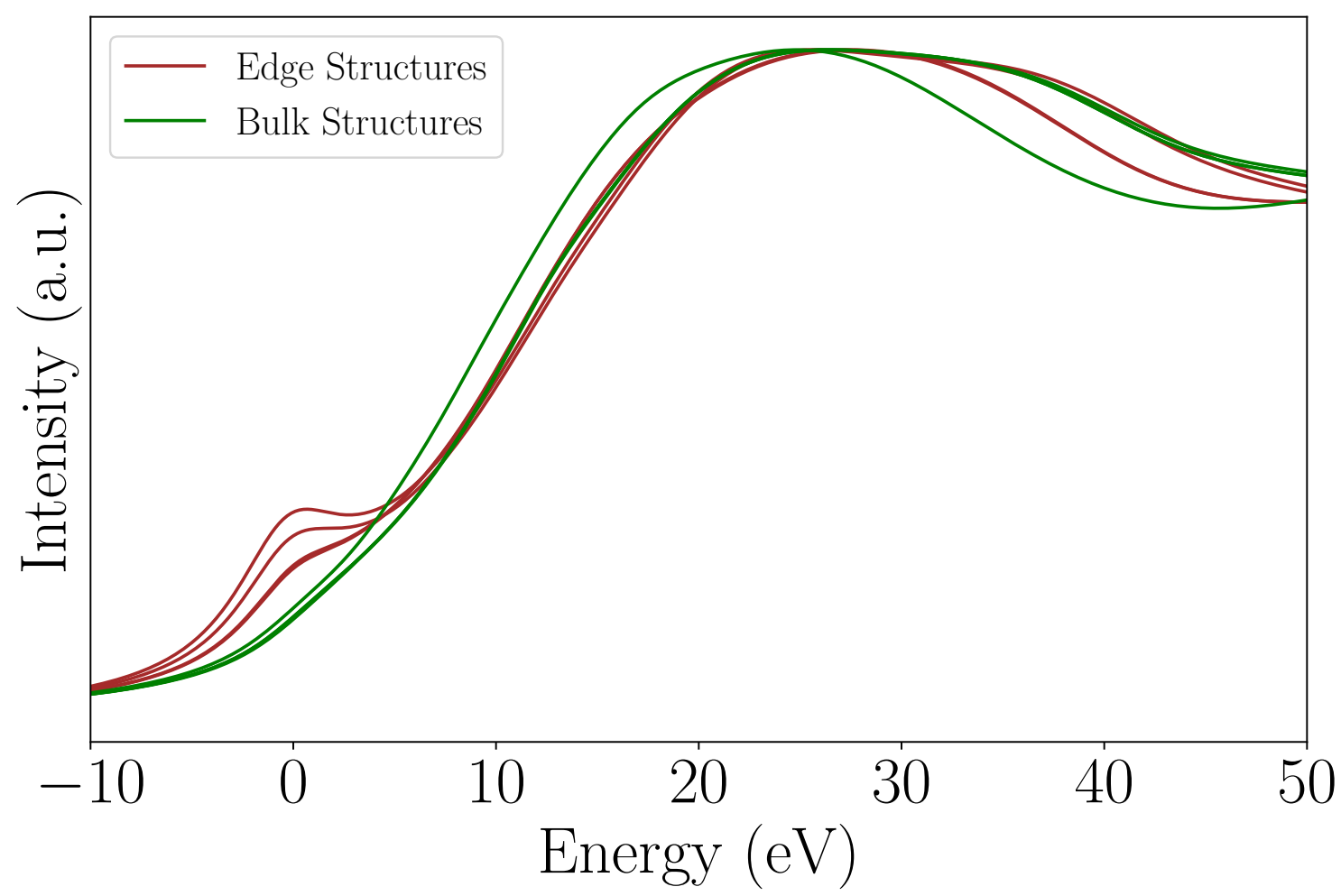

Figure S7: Pd *K*-edge calculated using the muffin-tin approximation for edge and bulk structures

Pd-N-C Pd K-edge XANES

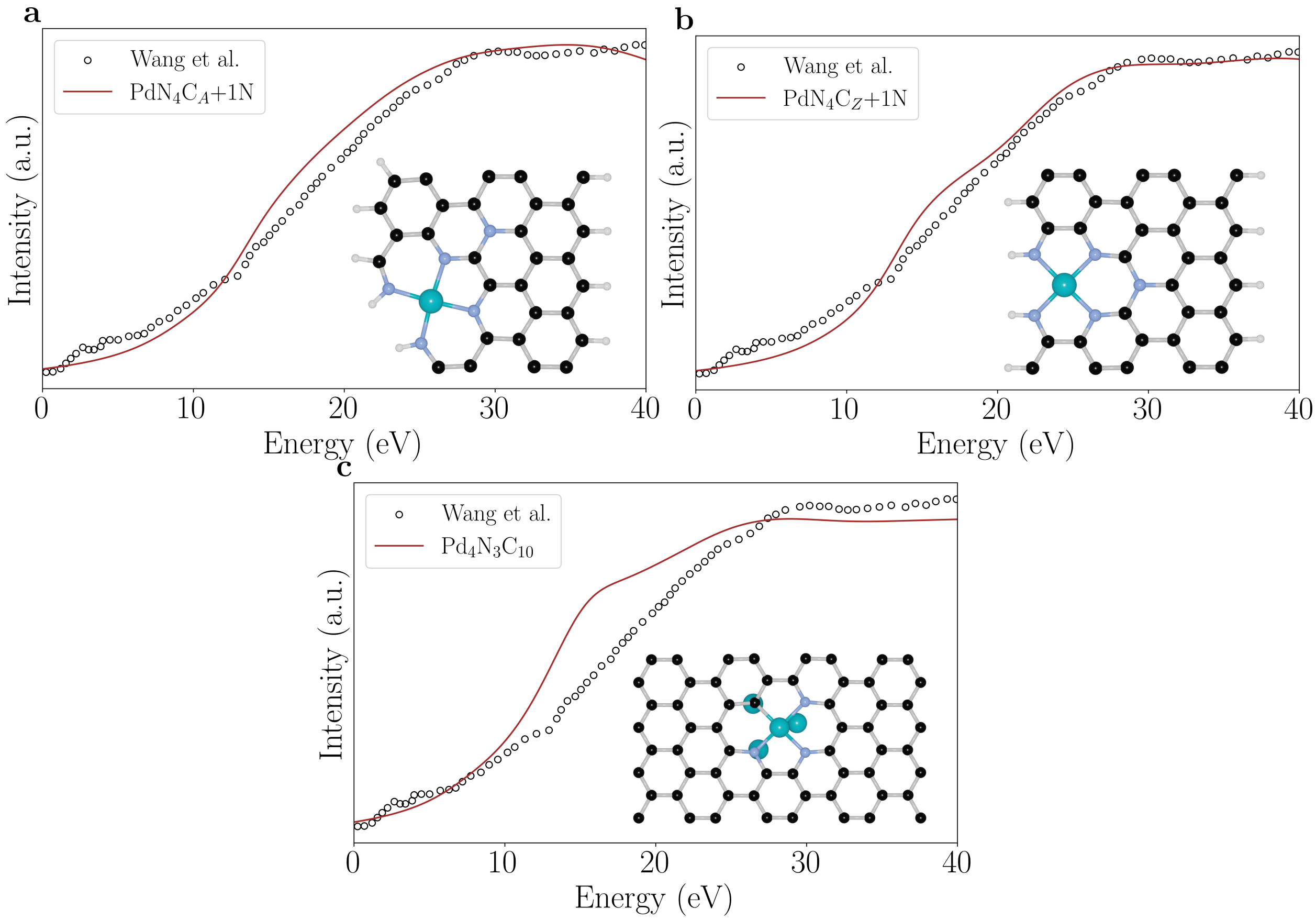

Figure S8: Calculated Pd K-edge XANES (red) with reference to Wang et al. experimental PdNC K-edge XANES spectrum (black dots).[2] The R-factor given quantifies the goodness of fit of each calculated spectrum with experiment. (Inset) Atomic scale structure; green=Pd, blue=N, black=C and white=H

Functional testing XPS small molecules

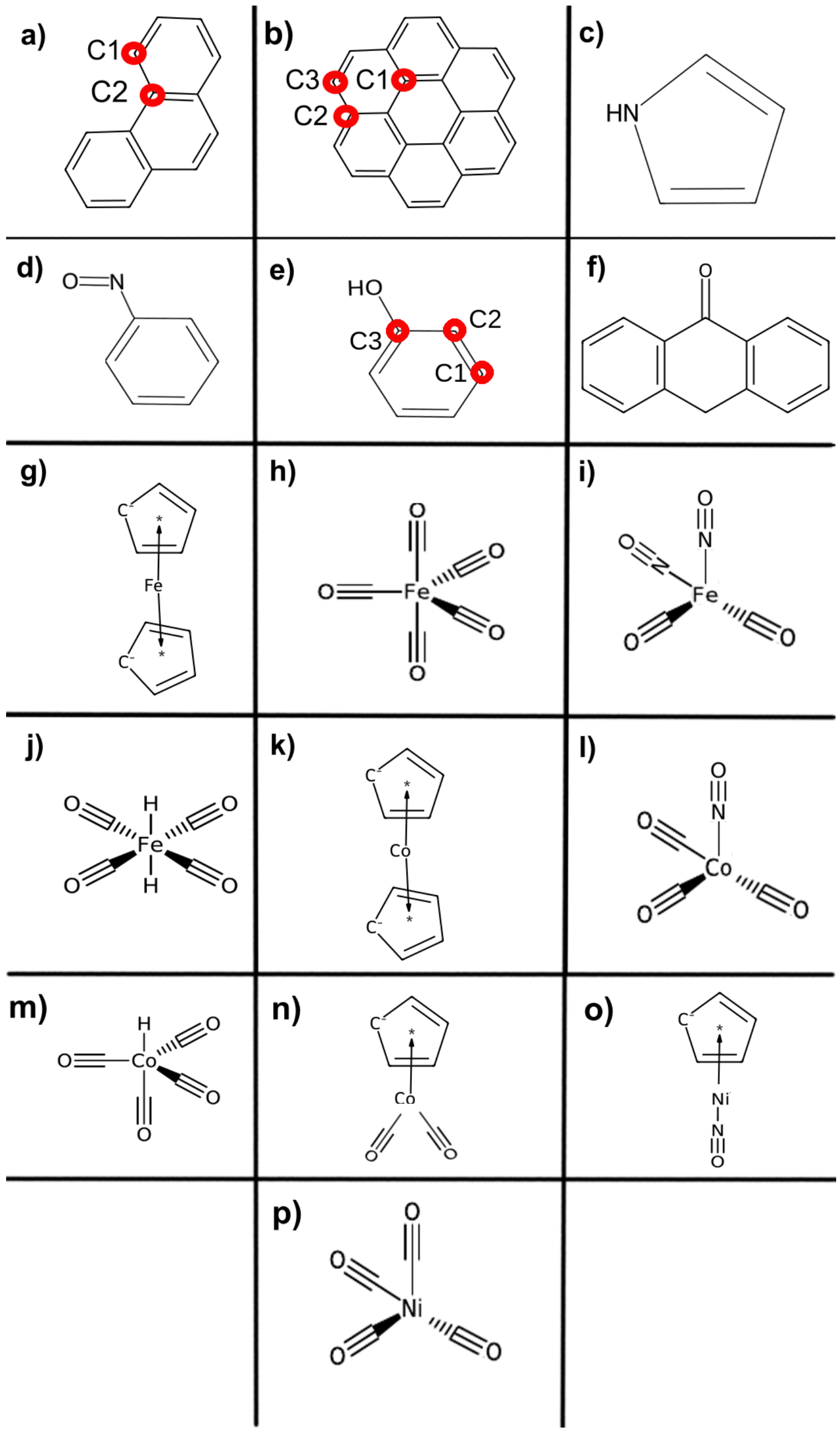

Figure S9: Molecules studied for XPS testing; a) phenanthrene, b) coronene, c) pyrrole, d) nitrosobenzene, e) phenol, f) anthrone, g) ferrocene, h) iron pentacarbonyl i) iron nitrosocarbonyl, j) iron tetracarbonyl dihydride, k) cobaltocene, l) cobalt tricarbonyl nitrosyl, m) cobalt tetracarbonyl hydride, n) cyclopentadienylcobalt dicarbonyl, o) cyclopentadienyl nickel nitrosyl and p) tetracarbonylnickel. Each red circle refers to a carbon atom in Table 1

Table 1: Calculated gaseous core-level binding energies ($-\frac{1}{3} \times$ spin-orbit coupling for transition metals) given alongside experimental gas phase XPS values of small molecules using B3LYP, PBE0 and PBE. Alphabetical labels reference structures in Fig. S9. All values given in eV.

	Formula	core-level // label	Exp.	B3LYP	PBE0	PBE
a)	$C_{14}H_{10}$	C 1s // C1	289.9[3]	289.9	289.5	289.1
		C 1s // C2	290.2[3]	290.2	289.8	289.4
b)	$C_{24}H_{12}$	C 1s // C3	289.6[3]	289.6	289.3	288.9
		C 1s // (C1,C2)	289.9[3]	289.8	289.6	289.2
c)	C_4H_4NH	N 1s // N	406.3[4]	406.3	406.0	405.6
d)	C_6H_5NO	N 1s // N	407.8[4]	407.7	407.4	406.6
		O 1s // O	538.7[4]	538.3	537.9	537.6
e)	C_6H_5OH	C 1s // C1	290.6[4]	290.4	290.0	289.6
		C 1s // C2	290.2[4]	290.2	289.8	289.4
		C 1s // C3	292.0[4]	292.1	291.7	291.3
		O 1s // O	538.9[4]	539.2	538.8	538.5
f)	$C_{14}H_{10}O$	C 1s // C-O	293.4[5]	291.9	291.5	291.0
		C* 1s // C-C	290.7[4]	290.1	289.8	289.4
		O 1s // O	536.8[5]	535.9	535.6	535.3
g)	$Fe(C_5H_5)_2$	Fe $2p_{3/2}$ // Fe	713.1[4]	712.4	712.5	711.0
		C 1s // C	290.0[4]	290.0	289.7	289.3

h)	$Fe(CO)_5$	Fe $2p_{3/2}$ // Fe	715.8[4]	714.8	714.8	713.6
		C 1s // C	293.4[4]	294.5	294.1	293.3
		O 1s // O	540.5[4]	540.4	540.0	539.6
i)	$Fe(CO)_2(NO)_2$	Fe $2p_{3/2}$ // Fe	715.6[4]	714.8	714.7	713.5
j)	$H_2Fe(CO)_4$	Fe $2p_{3/2}$ // Fe	716.0[4]	715.1	715.2	713.8
		C 1s // C	294.2[4]	294.8	294.4	293.5
		O 1s // O	540.2[4]	540.6	540.2	539.7
k)	$Co(C_5H_5)_2$	Co $2p_{3/2}$ // Co	784.8[4]	783.6	783.7	782.2
		C 1s // C	290.0[4]	289.7	289.3	288.9
l)	$Co(CO)_3NO$	Co $2p_{3/2}$ // Co	786.9[4]	786.1	785.9	785.0
		C 1s // C	293.8[4]	294.2	293.8	293.0
		N1s // N	407.8[4]	407.7	408.4	407.7
		O 1s // O-C	540.0[4]	540.2	539.8	539.4
m)	$HCo(CO)_4$	Co $2p_{3/2}$ // Co	786.9[4]	786.4	786.4	785.2
		C 1s // C	293.9[4]	294.5	294.2	293.3
		O 1s // O	540.1[4]	540.6	540.2	539.8
n)	$Co(C_5H_5)(CO)_2$	Co $2p_{3/2}$ // Co	786.3[4]	786.4	786.4	783.4
		C 1s // C-O	292.7[4]	293.0	292.6	291.9
		O 1s // O	539.0[4]	539.2	538.8	538.4

o)	$Ni(C_5H_5)NO$	Ni $2p_{3/2}$ // Ni	861.3[4]	860.1	859.9	858.8
		N1s // N	407.3[4]	406.9	407.3	406.4
		O 1s // O	539.2[4]	538.9	538.5	538.4
		C 1s // C	290.4[4]	290.6	290.2	289.8
p)	$Ni(CO)_4$	Ni $2p_{3/2}$ // Ni	861.2[4]	860.6	860.4	859.7
		C 1s // C	293.8[4]	294.3	293.8	293.1
		O 1s // O	540.1[4]	540.3	539.9	539.6

Table 2: Statistics of calculated vs. experimental core-level binding energies where MAE = Mean Absolute Error, ME = Mean Error and n is the test set size.

Functional	MAE (eV)	ME (eV)	$\boldsymbol{n}$
	**C 1s**		
B3LYP	0.4	0.1	18
PBE0	0.4	-0.3	18
PBE	0.9	-0.9	18
	**N 1s**		
B3LYP	0.1	-0.1	4
PBE0	0.4	0.0	4
PBE	0.7	-0.7	4
	**O 1s**		
B3LYP	0.3	0.0	10
PBE0	0.4	-0.4	10
PBE	0.7	-0.7	10
	**Fe $2p_{3/2}$**		
B3LYP	0.8	-0.8	4
PBE0	0.8	-0.8	4
PBE	2.2	-2.2	4
	**Co $2p_{3/2}$**		
B3LYP	1.0	-1.0	4
PBE0	1.1	-1.1	4
PBE	2.3	-2.7	4
	**Ni $2p_{3/2}$**		
B3LYP	0.9	-0.9	2
PBE0	1.1	-1.1	2
PBE	2.0	-2.0	2

Pd-N-C Extrapolated ΔSCF Convergence Plots

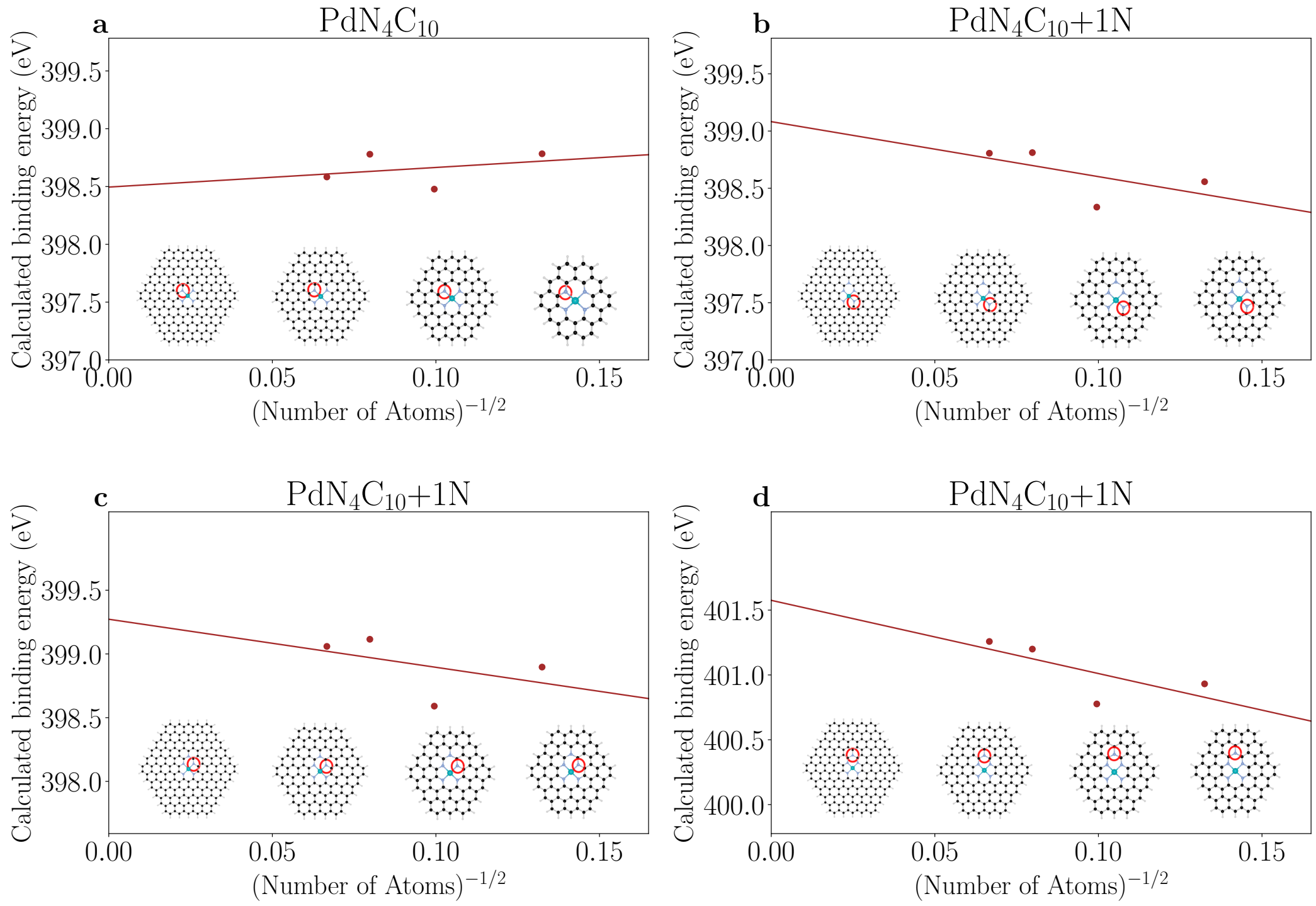

Figure S10: Extrapolated VBM ΔSCF method convergence plots for increasing size cluster models are used to calculate absolute N1s XPS binding energies for Pd-N-C active sites

Sensitivity Analysis

A sensitivity analysis is given for perturbations in the binding energies, line shape and peak width in Table 3. The best fit Gaussian peak is given for reference also using a σ taken from the experimental N1s spectrum. The maximum ± 0.1 eV error in XPS binding energies is taken from the mean absolute error in the main text Table 1. The Voigt line-shape fitting is given in Fig. S11.

Table 3: Sensitivity analysis of the PdN_4C_{10} and PdN_4C_{10}+1N distributions. The quoted Gaussian reference distribution is calculated using a σ taken from the experimental N1s spectrum

	**PdN_4C_{10} (%)**	**PdN_4C_{10}+1N (%)**	**R^2**
**Reference**			
Gaussian, $\sigma = 0.58$ eV	59	41	0.9830
**Binding-energy perturbation**			
All peaks systematically shifted, -0.05 eV	60	40	0.9848
All peaks systematically shifted, $+0.05$ eV	56	44	0.9804
All peaks systematically shifted, -0.1 eV	60	40	0.9859
All peaks systematically shifted, $+0.1$ eV	51	49	0.9769
Peaks independently shifted, ± 0.1 eV	75-17	25-83	0.9903/0.9746
**Line shape**			
Voigt, total FWHM fixed at 1.37 eV ($\gamma = 0.128$ eV)	62	38	0.9914
**Peak width**			
$\sigma = 0.52$ eV (-10%)	54	46	0.9641
$\sigma = 0.64$ eV ($+10\%$)	67	33	0.9924
**Combined perturbation**			
All four peaks -0.1 eV, $\sigma = 0.52$ eV (-10%)	56	44	0.9712
All four peaks -0.1 eV, $\sigma = 0.64$ eV ($+10\%$)	66	34	0.9930
All four peaks $+0.1$ eV, $\sigma = 0.52$ eV (-10%)	47	53	0.9511
All four peaks $+0.1$ eV, $\sigma = 0.64$ eV ($+10\%$)	64	36	0.9904

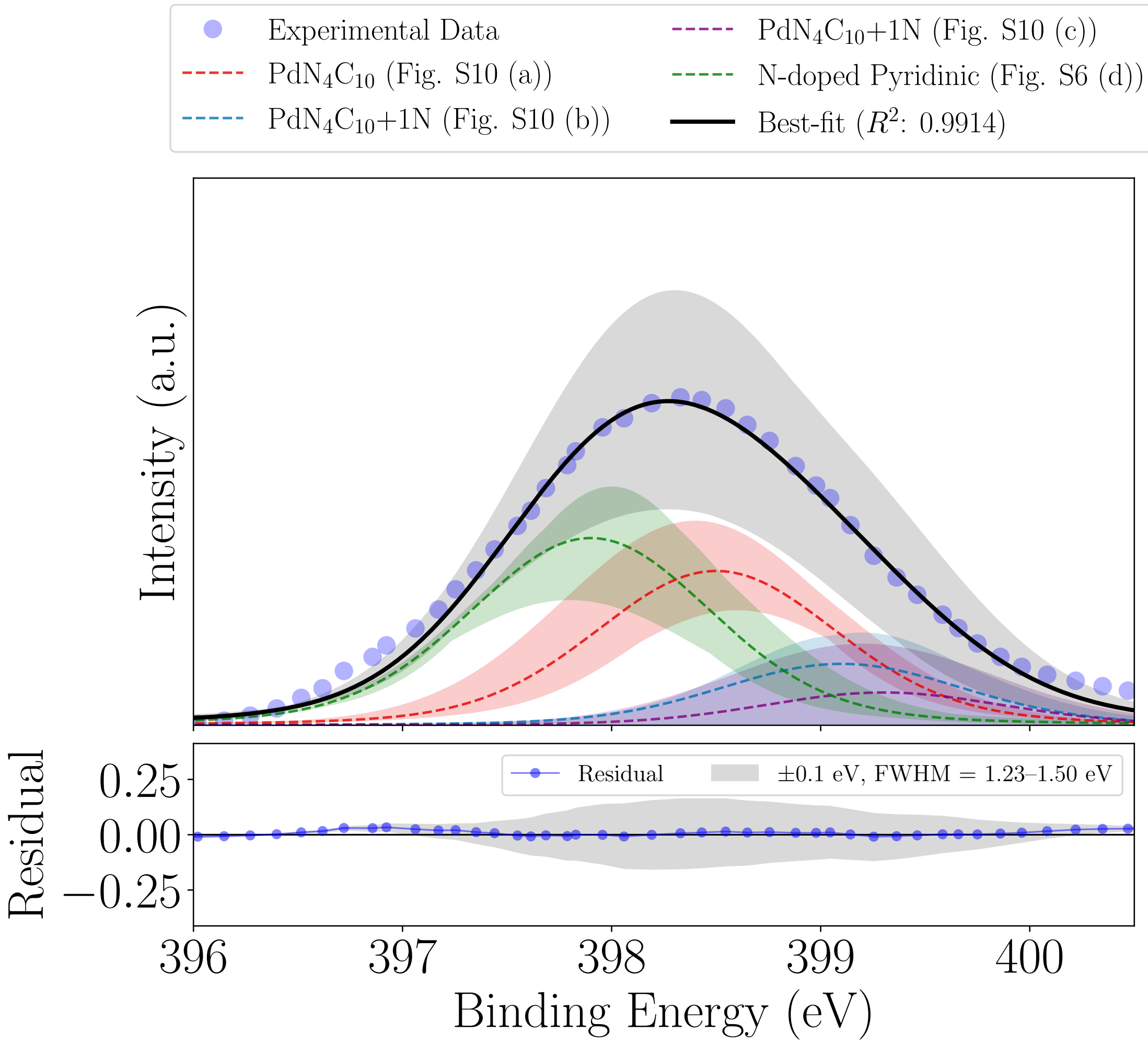

Figure S11: (top panel) Deconvoluted and Shirley background corrected experimental N1s XPS pyridinic peak[2] (blue dots). The best-fit summation (black curve) of each individual active site Voigt peak is optimised until a satisfactory agreement with experiment is found. The shaded areas are the deviation from the best-fit after applying a systematic error of $\pm$ 0.1 eV to all peak binding energies and a $\pm$ 10% perturbation to the peak width. The gray shaded area is the result of combining the errors of individual peaks. The figure label in parenthesis shows where the absolute XPS value is calculated using the extrapolated ΔSCF procedure. The experimental spectrum has been corrected using a Shirley background correction[6] (bottom panel) Residuals of the best-fit (circles) and the errors (gray) of the summed peak

Additional Microkinetic Modelling Curves

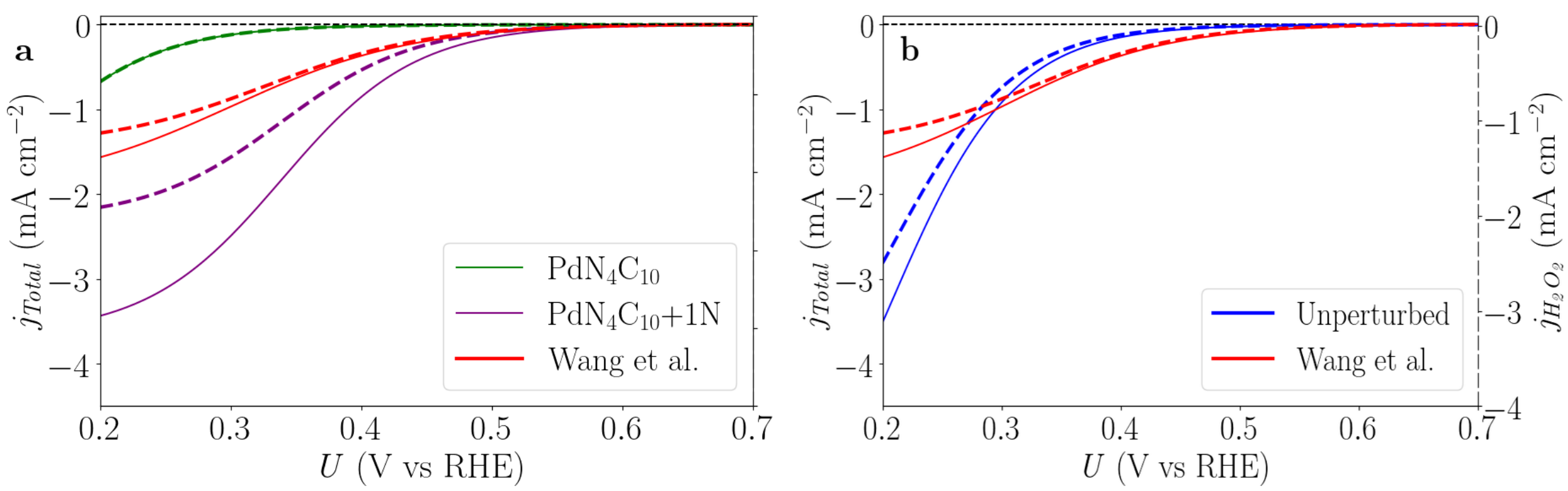

Figure S12: Comparison of total calculated (solid line measured using the left y-axis) and partial H_2O_2 current (dashed line measured using the right y-axis); a) of the unperturbed individual active sites and b) shows the summation of active sites after applying the computed distribution. Both left) and right) reference Wang et al. experiment.[2] All curves are shown at 1600 rpm

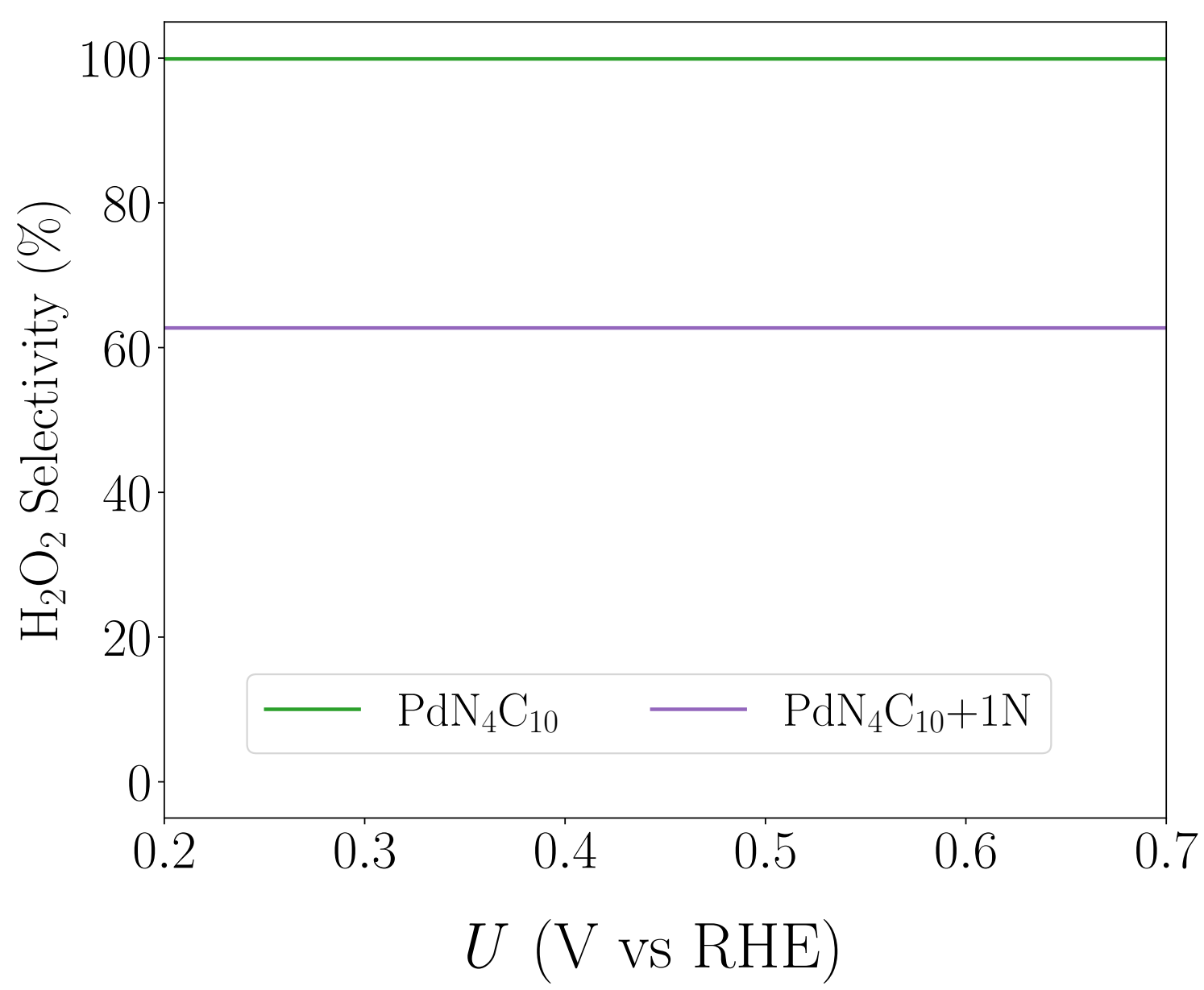

Figure S13: H_2O_2 selectivity for PdN_4C_{10} and PdN_4C_{10}+1N active sites

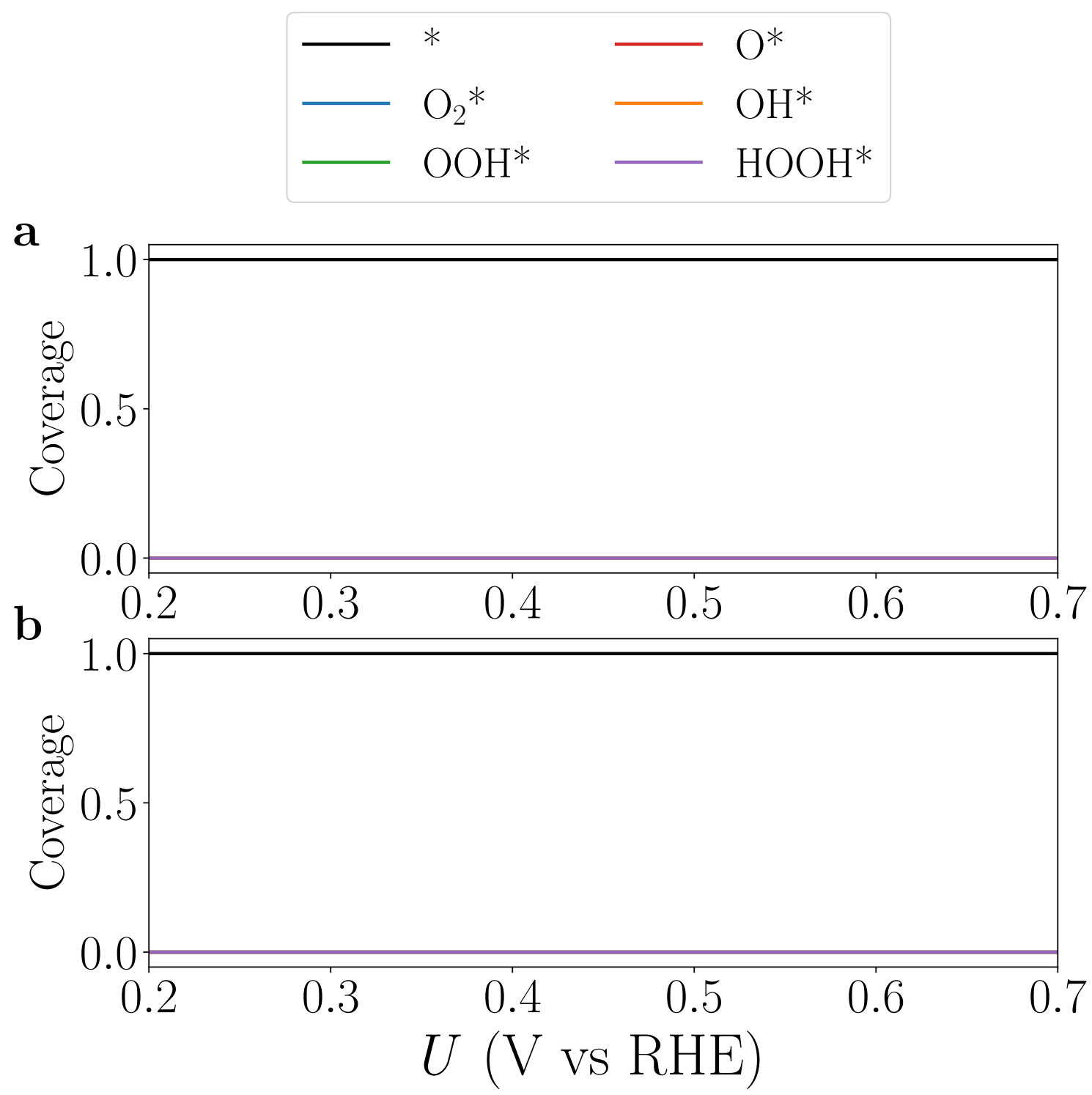

Figure S14: Coverages of adsorbates of a) PdN_4C_{10} and b) PdN_4C_{10}+1N active sites

Computational Details

The FDMNES program was used to calculate each XANES spectrum using the finite-differences method with a converged cut-off radius of 6Å. Quadrupolar transitions are included in each calculation, which are important for K-edge spectra. A Hedin and Lundqvist[7] exchange-correlation potential was used. A range of -10 eV below the Fermi level up to 100 eV was sampled at an interval of 0.1 eV. The results are convolved by an arctangent function to obtain the final spectrum for comparison with experiment. The calculated spectrum was shifted to align the first-derivative maximum with that of the experimental spectrum.

All ΔSCF calculations were performed using the B3LYP[8] functional implemented in the ADF program, with scalar relativistic effects accounted for using the scaled Zero-Order Regular Approximation (ZORA) method.[9,10] An SCF convergence of 10^{-8} was used. A triple zeta basis set plus an extra polarisation (TZP) function was used for all atoms not probed by XPS, while an even-tempered basis with quadruple zeta character plus an extra

three polarisation functions (ET-QZ3P) was used for the atoms probed by XPS. Unrestricted calculations were performed for open shell systems. The convergence of PdNC edge structure core-hole calculations was drastically improved by imposing a negative charge that prevents charge sloshing.

Spin-polarised adsorbate calculations were performed using the Vienna Ab Initio Simulation Package (VASP)[11–13] utilizing the projector-augmented wave (PAW)[14] method to describe core electrons. The valence electrons were modelled using a plane-wave basis set with an energy cut-off of 600 eV. A Fermi-smearing scheme of width 0.1 eV was used. The BEEF-vdW[15] exchange-correlation functional was used alongside an SCF convergence of 10^{-5} eV. Structural relaxation of structures were performed, ensuring the max norm of the force are below 0.025 eV/Å. The Atomic Simulation Environment (ASE)[16] package was used to construct the periodic models. The unit cell dimensions average 16.9×9.8 Å in the catalyst plane. Dipole corrections were applied perpendicular to the catalyst plane to decouple the electrostatic dipole interaction between images. The Brillouin zone was sampled using a $3 \times 3 \times 1$ Monkhorst-Pack k-point grid. For binding energy calculations, solvent effects were incorporated via the implicit solvent model in VASPsol[17,18] using a dielectric constant of 78.4 for water. To calculate thermodynamic free energies (ΔG) of adsorbates, zero-point energy (ZPE), vibrational internal energy (U_{vib}), and vibrational entropy (S_{vib}) corrections were computed using the "Vibrations" class within the thermochemistry module in ASE, and added to a DFT calculated energy.

Bulk and Edge N-doped System Thermodynamics

The difference in stoichiometries was accounted for by incorporating the chemical potentials of carbon and hydrogen. The chemical potential of carbon, μ_{C}, was calculated from graphene, while the chemical potential of hydrogen, μ_{H}, was calculated from the hydrogen molecule.

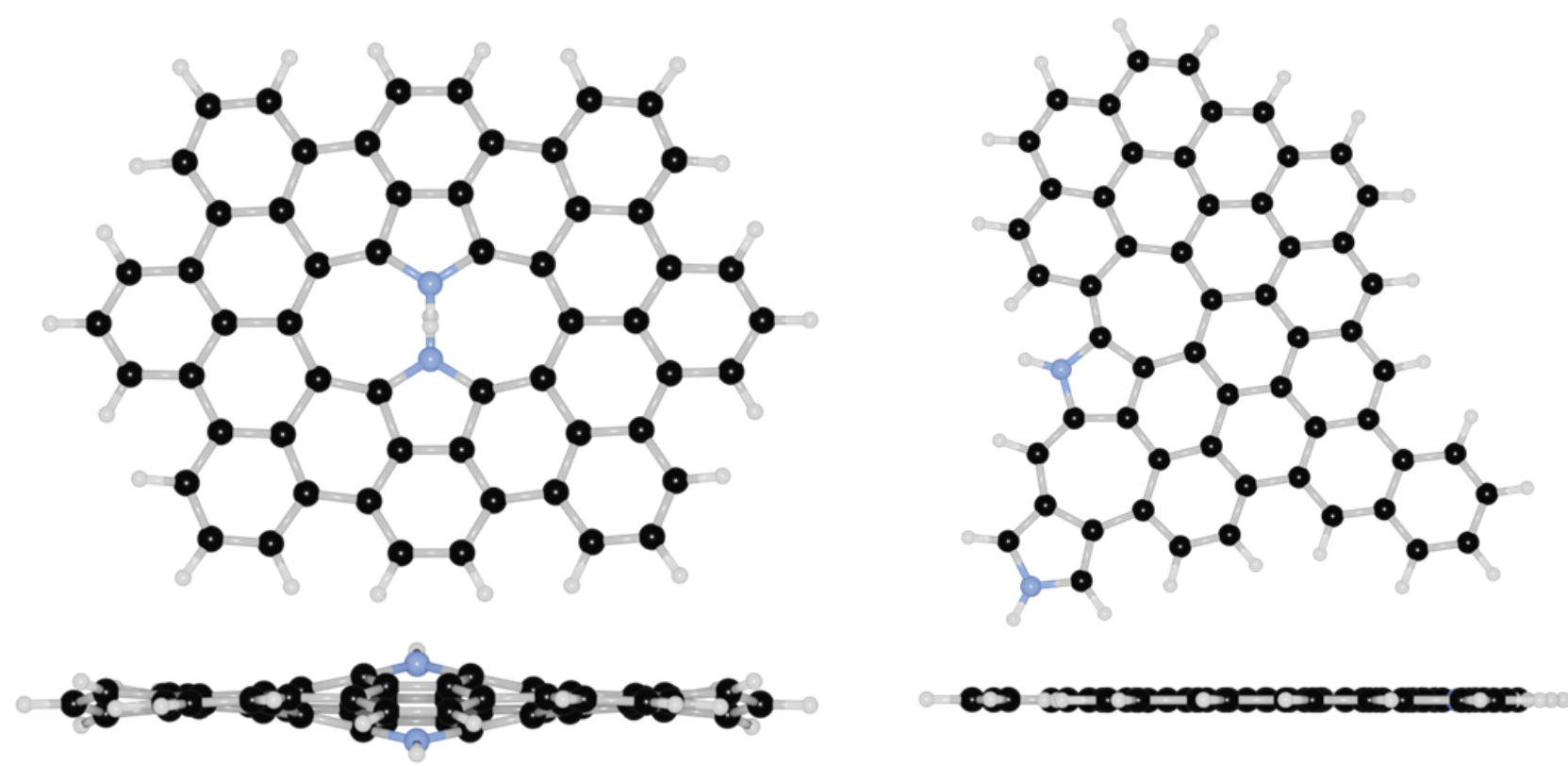

Figure S15: Left) Top down and side view of bulk $C_{58}N_2H_{24}$. Right) Top down and side view of edge $C_{57}N_2H_{23}$

The chemical potential of carbon is defined as the total energy per atom in graphene

$$\mu_{\mathrm{C}} = \frac{E_{\mathrm{graphene}}}{N_{\mathrm{C}}} \tag{1}$$

where E_{graphene} is the total energy of a graphene system containing N_{C} carbon atoms.

Similarly, the chemical potential of hydrogen is calculated from the total energy of the hydrogen molecule per hydrogen atom

$$\mu_{\mathrm{H}} = \frac{1}{2}E_{\mathrm{H_2}} \tag{2}$$

where $E_{\mathrm{H_2}}$ is the total energy of a H_2 molecule.

Adsorption Thermodynamics

Due to poor description of O_2 by DFT, the free energy of water formation (four electron pathway) is defined in accordance with the experimental free energy of liquid water formation

$$2H_{2(g)} + O_{2(g)} \rightarrow 2H_2O_{(l)}, \quad \Delta G_{H_2O} = -4.92\,\mathrm{eV}. \tag{3}$$

The Gibbs free energy of formation of H_2O_2 is defined using the experimental free energy

of formation of liquid H_2O_2:

$$H_{2(g)} + O_{2(g)} \rightarrow H_2O_{2(l)}, \quad \Delta G_{H_2O2} = -1.36\,\text{eV}. \tag{4}$$

The adsorption energies of all intermediates were defined relative to H_2O and H_2, with Christensen corrections[19,20] applied to the BEEF-vdW functional; +0.09 eV for H_2, and −0.03 eV for H_2O. Additionally, ΔG(OOH*) added a Christensen correction ΔE(O-O) = 0.20 eV.

The applied potential (U) was modelled in an approximate manner using the computational hydrogen electrode (CHE)[21,22] model. Furthermore, the free energy of a proton-electron pair was defined as (-eU) relative to gas phase H_2 with respect to the reversible hydrogen electrode (RHE). This gives the following electrochemical relation of the DFT free energy at zero voltage (ΔG_i^0) to an applied voltage (ΔG_i) for step i as

$$\Delta G_i = \Delta G_i^0 + neU \tag{5}$$

where n is the number of electrons in each reaction, e is the charge of the electron, k_B is the Boltzmann's constant and T is the temperature. A pH = 0 was used in all calculations.

The two-electron associative pathway is described using the following reaction scheme;

$$\mathrm{O_2}(g) + \mathrm{H^+} + e^- \rightarrow \mathrm{OOH^*} \quad ; \quad \Delta G_1 \tag{6}$$

$$\mathrm{OOH^*} + \mathrm{H^+} + e^- \rightarrow \mathrm{H_2O_2(l)} \quad ; \quad \Delta G_2 \tag{7}$$

with the applied potential dependence of ΔG adjusted using the CHE. The limiting potential for the two electron pathway is given by

$$U_L^{O_2/H_2O_2} = -max\{\Delta G_1, \Delta G_2\}/e \tag{8}$$

with the overpotential (U_η) given as a difference between the equilibrium potential and $U_L^{O_2/H_2O_2}$.

$$U_\eta^{O_2/H_2O2} = 0.68V - U_L^{O_2/H_2O2} \tag{9}$$

Analogously, the four-electron pathway is given by

$$\mathrm{O_2}(g) + \mathrm{H}^+ + e^- \rightarrow \mathrm{OOH}^* \quad ; \quad \Delta G_1 \tag{10}$$

$$\mathrm{OOH}^* + \mathrm{H}^+ + e^- \rightarrow *\mathrm{O} + \mathrm{H_2O(l)} \quad ; \quad \Delta G_2 \tag{11}$$

$$\mathrm{O}^* + \mathrm{H}^+ + e^- \rightarrow \mathrm{OH}^* \quad ; \quad \Delta G_3 \tag{12}$$

$$\mathrm{OH}^* + \mathrm{H}^+ + e^- \rightarrow * + \mathrm{H_2O(l)} \quad ; \quad \Delta G_4 \tag{13}$$

with the four electron limiting potential $U_L^{O_2/H_2O}$ given as

$$U_\eta^{O_2/H_2O} = 1.23V - U_L^{O_2/H_2O} \tag{14}$$

The dissociative mechanism is not considered as the dissociation barrier of OOH^* and $O_2{}^*$ on the active site centre coordinated with a carbon site has been shown to be prohibitively large for stronger binding Fe-N-C.[23]

Microkinetic Model

More information on the microkinetic model can be found in a previous paper[24], but a brief synopsis will be given here.

To begin, all steps below are incorporated into the model

$$O_2(\text{aq}) \underset{k_{-1}}{\overset{k_1}{\rightleftharpoons}} O_2(\text{dl}) \tag{15}$$

$$O_2(\text{dl}) + * \underset{k_{-2}}{\overset{k_2}{\rightleftharpoons}} O_2* \tag{16}$$

$$O_2 * + H^+ + e^- \underset{k_{-3}}{\overset{k_3}{\rightleftharpoons}} OOH* \tag{17}$$

$$OOH * + H^+ + e^- \underset{k_{-4}}{\overset{k_4}{\rightleftharpoons}} O * + H_2O \tag{18}$$

$$O * + H^+ + e^- \underset{k_{-5}}{\overset{k_5}{\rightleftharpoons}} OH* \tag{19}$$

$$OH * + H^+ + e^- \underset{k_{-6}}{\overset{k_6}{\rightleftharpoons}} * + H_2O \tag{20}$$

$$OOH * + H^+ + e^- \underset{k_{-7}}{\overset{k_7}{\rightleftharpoons}} H_2O_2* \tag{21}$$

$$H_2O_2* \underset{k_{-8}}{\overset{k_8}{\rightleftharpoons}} * + H_2O_2 \tag{22}$$

with (15) modelled using a one-dimensional diffusion model derived from Fick's first law at steady state. (16) and (22) are treated as chemical steps with the rate constants k_i derived from transition state theory

$$k_i = \nu_i exp\left(-\frac{G_{a,i}}{k_B T}\right) \tag{23}$$

$$G_{a,i} \approx E_{a,i} + \Delta G_i \tag{24}$$

where ν_i is the pre-exponential factor including solvent reorganisation effects at the electrode interface.[25] These chemical steps are assumed to be barrierless hence

$$G_{a,i} \approx \Delta G_i \tag{25}$$

For all remaining electrochemical reduction steps, the rate constant takes the form

$$k_i = A_i exp\left(-\frac{E^0_{a,i}}{k_B T}\right) exp\left(\frac{e\beta_i(U - U^0_i)}{k_B T}\right) \tag{26}$$

and A_i is a pre-factor on the timescale of solvent reorganisation for proton-coupled electron transfers and U is the operating potential.[26] U^0_i is the reversible potential of step i

$$U^0_i = \frac{-\Delta G^0_i}{e} \tag{27}$$

where ΔG^0_i is the reaction free energy at 0 V. For each reduction step, a previous DFT study[27] suggests $\beta_i = 0.5$ and $E^0_{a,i} = 0.26$ eV as a suitable Brønsted-Evans-Polanyi (BEP) slope and activation energy barrier at equilibrium respectively. This BEP relation essentially relates the reaction energy of each step to the activation energy required to reach the transition state. Also the activation entropy is included in A_i and is taken to be independent of the site. A proton transfer barrier at equilibrium of 0.26 eV has been used with previous microkinetic modelling studies of Pd single atom catalyst structures[28]. These studies build on the seminal computational work of Tripković et al.[27] and the experimental investigation by Dubey et al.,[29] which established this value for oxygen-based intermediates involved in

electrocatalytic reactions.

Using k_i and their respective backward rate constants k_{-i} related through the equilibrium constant (K_i), one can construct a set of coupled ordinary differential equations as given

$$\frac{\partial x_{O_2(\mathrm{dl})}}{\partial t} = k_1 X_{O_2(\mathrm{aq})} - k_{-1} x_{O_2(\mathrm{dl})} - k_2 x_{O_2(\mathrm{dl})}\theta_* + k_{-2}\theta_{O_2*} \tag{28}$$

$$\frac{\partial \theta_*}{\partial t} = k_6\theta_{OH*} - k_{-6}\theta_* x_{H_2O} - k_2 x_{O_2(\mathrm{dl})}\theta_* + k_{-2}\theta_{O_2*} + k_8\theta_{H_2O_2*} - k_{-8}\theta_* x_{H_2O_2} \tag{29}$$

$$\frac{\partial \theta_{O_2*}}{\partial t} = k_2 x_{O_2(\mathrm{dl})}\theta_* - k_{-2}\theta_{O_2*} - k_3\theta_{O_2*} + k_{-3}\theta_{OOH_*} \tag{30}$$

$$\frac{\partial \theta_{OOH_*}}{\partial t} = k_3\theta_{O_{2*}} - k_{-3}\theta_{OOH_*} - k_4\theta_{OOH*} + k_{-4}x_{H_2O}\theta_{O*} - k_7\theta_{OOH*} + k_{-7}\theta_{H_2O2*} \tag{31}$$

$$\frac{\partial \theta_{O^*}}{\partial t} = k_4\theta_{OOH^*} - k_{-4}x_{H_2O}\theta_{O^*} - k_5\theta_{O^*} + k_{-5}\theta_{OH^*} \tag{32}$$

$$\frac{\partial \theta_{OH^*}}{\partial t} = k_5\theta_{O^*} - k_{-5}\theta_{OH^*} - k_6\theta_{OH^*} + k_{-6}\theta_* x_{H_2O} \tag{33}$$

$$\frac{\partial \theta_{H_2O_2*}}{\partial t} = k_7\theta_{OOH_*} - k_{-7}\theta_{H_2O_2*} - k_8\theta_{H_2O_2*} + k_{-8}\theta_* x_{H_2O_2} \tag{34}$$

where x is the mole fraction of the species, θ is coverage of the species, and t is time. $x\mathrm{H_2O}$ is taken as 1, and $x\mathrm{O}_{2(aq)}$ is taken as 2.3410×10^{-5} representing $\mathrm{O}_{2(g)}$ in equilibrium with $\mathrm{O}_{2(aq)}$ at 1 atm.

Furthermore, site conservation must be satisfied

$$1 = \theta_* + \theta_{\mathrm{O_2*}} + \theta_{\mathrm{OOH*}} + \theta_{\mathrm{O*}} + \theta_{\mathrm{OH*}} + \theta_{\mathrm{H_2O_2*}} \tag{35}$$

Once solved to steady state, partial current densities of water (j_{H_2O}) and $\mathrm{H_2O_2}$ ($j_{H_2O_2}$) can be calculated

$$j_{H_2O} = -2e\rho TOF_{H_2O} \tag{36}$$

$$j_{H_2O2} = -2e\rho TOF_{H_2O2} \tag{37}$$

where ρ is the surface density of active sites comparable to Pt(111), $e\rho = 80.3\ \mu C/cm^2$,[30] and TOF_{H_2O} and $TOF_{H_2O_2}$ is the turnover frequency of H_2O and H_2O_2 respectively.

The rotating disk electrode rotation speed ω effect can be incorporated into k_1 via the following relation

$$k_1 = k_1^0 \sqrt{\frac{\omega}{\omega^0}} \tag{38}$$

where ω^0 is the rotation speed at 1600 rpm and k_1^0 is the k_1 rate constant adjusted to give a mass transport limited $4e^-$ current of 6 mA/cm^2 at 1600 rpm.

References

[1] J. M. Kahk, G. S. Michelitsch, R. J. Maurer, K. Reuter, J. Lischner, "Core Electron Binding Energies in Solids from Periodic All-electron Δ-Self-Consistent-Field Calculations", *J. Phys. Chem. Lett.* **2021**, *12*, 9353–9359.

[2] N. Wang, X. Zhao, R. Zhang, S. Yu, Z. H. Levell, C. Wang, S. Ma, P. Zou, L. Han, J. Qin, L. Ma, Y. Liu, H. L. Xin, "Highly Selective Oxygen Reduction to Hydrogen Peroxide on a Carbon-Supported Single-Atom Pd Electrocatalyst", *ACS Catal.* **2022**, *12*, 4156–4164.

[3] G. Fronzoni, O. Baseggio, M. Stener, W. Hua, G. Tian, Y. Luo, B. Apicella, M. Alfé, M. de Simone, A. Kivimäki, et al., "Vibrationally Resolved High-resolution NEXAFS and XPS Spectra of Phenanthrene and Coronene", *J. Chem. Phys.* **2014**, *141*, DOI `10.1063/1.4891221`.

[4] W. Jolly, K. Bomben, C. Eyermann, "Core-electron Binding Energies for Gaseous Atoms and Molecules", *At. Data Nucl. Data Tables* **1984**, *31*, 433–493.

[5] W. L. Jolly, T. F. Schaaf, "π-Donor relaxation in the Oxygen 1s Ionization of Carbonyl Compounds", *J. Am. Chem. Soc.* **1976**, *98*, 3178–3181.

[6] D. A. Shirley, "High-resolution X-ray Photoemission Spectrum of the Valence Bands of Gold", *Phys. Rev. B* **1972**, *5*, 4709.

[7] L. Hedin, B. I. Lundqvist, "Explicit Local Exchange-correlation Potentials", *J. Phys. C: Solid State Phys.* **1971**, *4*, 2064.

[8] P. J. Stephens, F. J. Devlin, C. F. Chabalowski, M. J. Frisch, "*Ab Initio* Calculation of Vibrational Absorption and Circular Dichroism Spectra Using Density Functional Force Fields", *J. Phys. Chem.* **1994**, *98*, 11623–11627.

[9] E. v. Lenthe, E.-J. Baerends, J. G. Snijders, "Relativistic Regular Two-component Hamiltonians", *J. Chem. Phys.* **1993**, *99*, 4597–4610.

[10] E. van Lenthe, E.-J. Baerends, J. G. Snijders, “Relativistic Total Energy Using Regular Approximations”, *J. Chem. Phys.* **1994**, *101*, 9783–9792.

[11] J. Furthmüller, J. Hafner, G. Kresse, “Dimer Reconstruction and Electronic Surface States on Clean and Hydrogenated Diamond (100) Surfaces”, *Phys. Rev. B* **1996**, *53*, 7334.

[12] G. Kresse, J. Furthmüller, “Efficiency of Ab-initio Total Energy Calculations for Metals and Semiconductors Using a Plane-Wave Basis Set”, *Comput. Mater. Sci.* **1996**, *6*, 15–50.

[13] G. Kresse, J. Furthmüller, “Efficient Iterative Schemes for *ab initio* Total-energy Calculations using a Plane-wave Basis Set”, *Phys. Rev. B* **1996**, *54*, 11169.

[14] G. Kresse, D. Joubert, “From Ultrasoft Pseudopotentials to the Projector Augmented-Wave Method”, *Phys. Rev. B* **1999**, *59*, 1758.

[15] J. Wellendorff, K. T. Lundgaard, A. Møgelhøj, V. Petzold, D. D. Landis, J. K. Nørskov, T. Bligaard, K. W. Jacobsen, “Density Functionals for Surface Science: Exchange-Correlation Model Development with Bayesian Error Estimation”, *Phys. Rev. B* **2012**, *85*, 235149.

[16] A. Hjorth Larsen, J. Jørgen Mortensen, J. Blomqvist, I. E. Castelli, R. Christensen, M. Dułak, J. Friis, M. N. Groves, B. Hammer, C. Hargus, E. D. Hermes, P. C. Jennings, P. Bjerre Jensen, J. Kermode, J. R. Kitchin, E. Leonhard Kolsbjerg, J. Kubal, K. Kaasbjerg, S. Lysgaard, J. Bergmann Maronsson, T. Maxson, T. Olsen, L. Pastewka, A. Peterson, C. Rostgaard, J. Schiøtz, O. Schütt, M. Strange, K. S. Thygesen, T. Vegge, L. Vilhelmsen, M. Walter, Z. Zeng, K. W. Jacobsen, “The Atomic Simulation Environment—a Python Library for Working with Atoms”, *J. Phys. Condens. Matter* **2017**, *29*, 273002.

[17] K. Mathew, R. Sundararaman, K. Letchworth-Weaver, T. Arias, R. G. Hennig, "Implicit Solvation Model for Density-functional Study of Nanocrystal Surfaces and Reaction Pathways", *J. Chem. Phys.* **2014**, *140*, 084106.

[18] K. Mathew, V. Kolluru, S. Mula, S. N. Steinmann, R. G. Hennig, "Implicit Self-Consistent Electrolyte Model in Plane-Wave Density-Functional Theory", *J. Chem. Phys.* **2019**, *151*, 234101.

[19] R. Christensen, H. A. Hansen, C. F. Dickens, J. K. Nørskov, T. Vegge, "Functional Independent Scaling Relation for ORR/OER Catalysts", *J. Phys. Chem. C* **2016**, *120*, 24910–24916.

[20] R. Christensen, H. A. Hansen, T. Vegge, "Identifying Systematic DFT Errors in Catalytic Reactions", *Catal. Sci. Technol.* **2015**, *5*, 4946–4949.

[21] J. K. Nørskov, J. Rossmeisl, A. Logadottir, L. Lindqvist, J. R. Kitchin, T. Bligaard, H. Jonsson, "Origin of the Overpotential for Oxygen Reduction at a Fuel-Cell Cathode", *J. Phys. Chem. B* **2004**, *108*, 17886–17892.

[22] A. Kulkarni, S. Siahrostami, A. Patel, J. K. Nørskov, "Understanding Catalytic Activity Trends in the Oxygen Reduction Reaction", *Chem. Rev.* **2018**, *118*, 2302–2312.

[23] Y. Wang, Y.-J. Tang, K. Zhou, "Self-adjusting Activity Induced by Intrinsic Reaction Intermediate in Fe-N-C Single-Atom Catalysts", *J. Am. Chem. Soc.* **2019**, *141*, 14115–14119.

[24] H. A. Hansen, V. Viswanathan, J. K. Nørskov, "Unifying Kinetic and Thermodynamic Analysis of 2 e^- and 4e^- Reduction of Oxygen on Metal Surfaces", *J. Phys. Chem. C* **2014**, *118*, 6706–6718.

[25] W.-P. Zhou, X. Yang, M. B. Vukmirovic, B. E. Koel, J. Jiao, G. Peng, M. Mavrikakis, R. R. Adzic, "Improving Electrocatalysts for O_2 Reduction by Fine-tuning the Pt-Support Interaction: Pt Monolayer on the Surfaces of a $Pd_3Fe(111)$ Single-crystal Alloy", *J. Am. Chem. Soc.* **2009**, *131*, 12755–12762.

[26] A. Kuzume, E. Herrero, J. M. Feliu, “Oxygen Reduction on Stepped Platinum Surfaces in Acidic Media”, *J. Electroanal. Chem.* **2007**, *599*, 333–343.

[27] V. Tripković, E. Skúlason, S. Siahrostami, J. K. Nørskov, J. Rossmeisl, “The Oxygen Reduction Reaction Mechanism on Pt(111) from Density Functional Theory Calculations”, *Electrochim. Acta* **2010**, *55*, 7975–7981.

[28] M. Rebarchik, S. Bhandari, T. Kropp, M. Mavrikakis, “How Noninnocent Spectator Species Improve the Oxygen Reduction Activity of Single-Atom Catalysts: Microkinetic Models from First-principles Calculations”, *ACS Catal.* **2020**, *10*, 9129–9135.

[29] M. K. Dubey, R. Mohrschladt, N. M. Donahue, J. G. Anderson, “Isotope Specific Kinetics of Hydroxyl Radical (OH) with Water (H_2O): Testing Models of Reactivity and Atmospheric Fractionation”, *J. Phys. Chem. A* **1997**, *101*, 1494–1500.

[30] J. Wang, N. Markovic, R. Adzic, “Kinetic Analysis of Oxygen Reduction on Pt(111) in Acid Solutions: Intrinsic Kinetic Parameters and Anion Adsorption Effects”, *J. Phys. Chem. B* **2004**, *108*, 4127–4133.
